# Two homologous sequences of Grp78 and HSP70 represent tumor antigens shared with streptococcal superantigens in eliciting an antitumor immune response: an immunoinformatic investigation

**DOI:** 10.3389/fimmu.2025.1644687

**Published:** 2025-09-11

**Authors:** Paola Finotti

**Affiliations:** Department of Pharmaceutical and Pharmacological Sciences, University of Padua, Padua, Italy

**Keywords:** heat shock proteins, tumor-associated antigens, bacterial antigens, superantigens, epitope mapping, immunodominant epitopes, computational biology, immunoinformatics

## Abstract

Bacteria-based therapies have gained increasing attention as novel immunotherapeutic approaches against tumors. Among them, bacteria producing superantigen (SAg) toxins are considered particularly effective due to their ability to induce potent, generalized inflammatory responses capable of destroying tumor cells. Building on evidence of the antitumor efficacy of certain streptococcal preparations, and on the known involvement of heat shock proteins (HSPs) in tumor progression, we tested the hypothesis that streptococcal SAgs may elicit an adaptive immune response against tumors by priming cytotoxic T cells with epitopes that closely resemble tumor-associated HSPs. Through a multistep immunoinformatic analysis, we identified HSP70, Grp78, and Grp94 as containing immunogenic epitopes with high similarity to those found in the SAg domains of streptococcal exotoxins. Notably, a long sequence of HSP70 and its homolog in Grp78 was found to harbor multiple immunodominant epitopes overlapping the MHC class I and II epitopes of exotoxins, also containing B-cell epitopes. Results suggest that specific sequences of HSP70 and Grp78 may act as shared tumor antigens targeted by the immune response initiated by streptococcal SAgs, supporting their potential use as peptide-based tumor vaccines.

## Introduction

Immunotherapy is currently one of the most promising strategies in cancer treatment, offering the potential for durable and complete remission and addressing key limitations of conventional therapies (1, 2). Its success depends on the ability to stimulate the immune system to mount an adaptive response capable of eliminating tumor cells without harming healthy tissue. This requires the identification of tumor-associated antigens (Ags) that activate T-cell subsets specifically targeting Ag-expressing tumor cells (3–6).

Within this context, bacterial immunotherapy has attracted renewed interest due to its intriguing and beneficial immunomodulatory effects (7–10). Since Coley’s early work in the late 1800s, where *Streptococcus pyogenes* preparations were used to treat bone sarcomas (11, 12), bacterial therapies have evolved and are now employed as adjuncts to standard treatments (13, 14) or as cancer vaccines, either alone or in combination with other tumor-targeting agents (15–18). Independent of their therapeutic formulation, bacteria have been shown to influence the outcomes of various cancer immunotherapies (19, 20). Notably, in esophageal squamous cell carcinoma, the abundance of *Streptococcus* in the intra-tumoral microbiome has been associated with improved response to chemoimmunotherapy (21).

Despite these advances, the precise mechanisms by which bacteria trigger effective *in vivo* antitumor immune responses remain incompletely understood. Certain bacterial strains, including *Streptococcus pyogenes* and *Staphylococcus aureus* (22, 23), produce potent protein toxins termed superantigens (SAgs), which induce extensive T-cell proliferation and cytokine release far exceeding the response elicited by conventional Ags (22, 24). While SAgs are also implicated in acute and chronic human diseases (24–27), their structural and functional characteristics, particularly a conserved SAg domain, suggest a shared capacity to induce robust inflammatory responses (22, 28).

The antitumor effects of bacterial therapies appear to be primarily mediated by this inflammation, which can occur both locally at the tumor site and systemically, potentially leading to tumor cell death independent of Ag specificity. Clinical observations dating back to Coley indicate that stronger febrile and inflammatory responses correlate with improved patient outcomes (11). Moreover, converting immunologically “cold” tumors into “hot,” inflamed tumors through pathogen-mimicking stimuli has been shown to improve responses to immunotherapy (29, 30). Acute infection-driven inflammation or exposure to bacterial/viral SAgs can also reshape the tumor microenvironment, promoting a transition from an immunosuppressive to a pro-inflammatory, cytotoxic-dominated antitumor response even at anatomical sites distant from the original immune activation (21, 31, 32).

Recent findings suggest that *Streptococcus* may directly colonize tumor tissues, promoting cytotoxic T-cell infiltration and enhancing immunotherapy responsiveness (21, 32). This raises the possibility that antitumor immunity may, in part, arise from Ag cross-reactivity between bacterial and tumor-derived molecules (31).

These insights have motivated the development of tumor-targeted SAg therapies, which aim to direct the immunostimulatory activity of bacterial toxins specifically to tumor sites (16, 18). Fusion constructs have been engineered combining SAgs with monoclonal antibodies or antibody fragments specific for tumor-associated Ags, allowing for localized cytokine release and tumor cell killing (33). Although promising results have been demonstrated *in vitro* (33) and in murine models (16, 34), clinical trials in humans have not achieved the desired outcomes (35, 36). This underscores the need to more precisely define how SAgs function within the complex immunological landscape of human cancers (31).

A retrospective analysis of Coley’s successes (11, 12, 37) suggests that intense inflammation alone may not fully account for tumor remission. An alternative mechanism could involve Ag-specific cytotoxic lymphocyte activation by streptococcal exotoxins, targeting tumor cells expressing similar Ags. If true, this implies molecular similarity between streptococcal exotoxins and tumor-associated Ags. Among the most plausible candidates are Heat Shock Proteins (HSPs), a conserved family of intracellular chaperones that when liberated from their physiological setting become potent inducers of both innate and Ag-specific immunity (38). HSPs are frequently overexpressed in tumors (39) and often exposed on the tumor cell surface or released into circulation, contributing to their strong immunogenicity (38, 40). HSP expression is closely linked to the oncogenic process, supporting tumor cell survival, proliferation, metastasis, chemoresistance, and poor clinical outcomes (41–43).

The most identified HSPs in tumors include Grp94 (40, 44), HSP90 (45, 46), Grp78 (47), HSP70 (46, 48), and HSP60 (49). Notably, many bacterial immunodominant Ags are themselves HSPs (50–52), and a high degree of sequence homology exists across species within each HSP family.

Based on this, we hypothesized that the antitumor activity of *Streptococcus pyogenes* SAgs may result from Ag-dependent T-cell activation driven by shared immunogenic peptides between SAgs and tumor-associated HSPs. To test this, we conducted an immunoinformatic analysis comparing major *Streptococcus pyogenes* exotoxins with human HSPs known to be tumor related. Our goal was to identify shared immunogenic epitopes that may serve as common Ags and potential targets for tumor-selective immunotherapy.

## Methods

### Protein retrieval

Protein sequences were retrieved from UniProtKB/Swiss-Prot (https://www.uniprot.org). The pyrogenic exotoxins of *Streptococcus pyogenes* (serotype M18) included: SPEA_STRP8 (P62561), SPEC_STRP8 (Q8NKX2), SPEM_STRPY (Q7WYA2), and SPEK_STRPY (A0A5S4TLM8). Human HSPs analyzed were: Endoplasmin (ENPL, P14625), HSP 90-alpha (P07900-1), Endoplasmic reticulum chaperone BiP (Grp78, P11021), HSP70 kDa 1A (HS71A, P0DMV8), and HSP60 (CH60, P10809). Tumor antigens retrieved were Trophoblast glycoprotein (TPBG_HUMAN, Q13641) and carcinoembryonic antigen-related cell adhesion molecule 5 (CEAM5_HUMAN, P06731-1).

### Methodological approach

**Figure 1:**
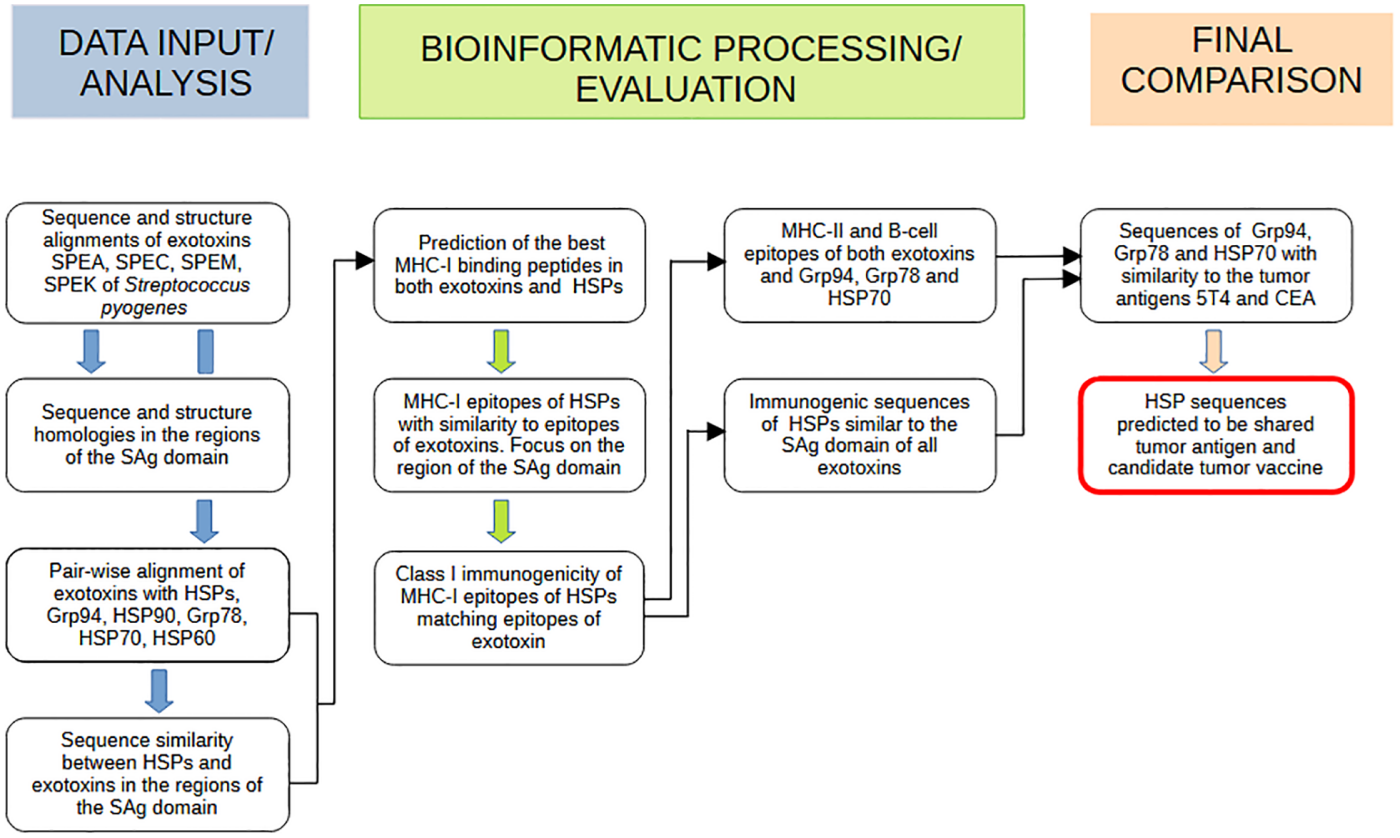
Flowchart outlining the strategy for identifying HSP sequences as shared tumor antigens. The process included key analytical phases to identify sequences in HSPs that share with *Streptococcus pyogenes* exotoxins the ability to activate immune responses. Each step summarizes the primary methods used to uncover HSP sequences with potential tumor antigen properties.

The methodology included a multi-step immunoinformatic analysis using common tools for designing epitope-based vaccines (53). As outlined in **Figure 1**, the first steps involved multiple sequence and structure alignments of exotoxins, and searching for sequence similarity between HSPs and exotoxins, focusing attention on the SAg region. The HSPs sequences with the best similarity with the common exotoxin region of SAg were analyzed for MHC-I, MHC-II, and B-cell epitopes. Only epitopes with positive class-I immunogenicity values were then considered for comparison with those identified in the SAg region of exotoxins. Matching epitopes would support the hypothesis that HSP sequences, structurally similar to SAgs, represent shared tumor antigens and potential targets of the exotoxin-driven antitumor response.

### Protein sequence and structure analysis

Protein sequences were analyzed in pairwise and multiple sequence alignments using T-Coffee (https://tcoffee.crg.eu), and Clustal Omega (https://www.uniprot.org/align). Although based on different algorithms, both programs yielded consistent results in cases of high sequence similarity. T-Coffee assigns a maximum consensus score of 1000 (100%). The Expresso function of T-Coffee (https://tcoffee.crg.eu/apps/tcoffee/do:expresso) was used to assess structural similarity. Only uninterrupted segments of at least eight amino acids with the highest consistency scores were considered. BLAST (https://blast.ncbi.nlm.nih.gov) was used to verify whether similar sequences were conserved among HSPs and between HSPs and tumor markers 5T4 and CEA. An expect threshold of E<0.05 was set to determine statistical significance.

### MHC class I, class II, and B-cell epitope detection

Predicted best MHC class I epitopes in exotoxins and HSPs were identified using the IEDB Analysis Resource (http://tools.iedb.org/mhci/), applying IEDB recommended 2020.09 methods: NetMHCpan EL 4.1predicting best MHC-I peptide score and percentile rank, and Ann 4.0 predicting IC50 values. A percentile rank of ≤0.5 and a IC50 value ≤500 nM were both considered indicative of strong binding (54). A representative panel of common HLA alleles was used: A*01:01; A*02:01; A*03:01; A*24:02; B*07:02; B*08:01; B*15:01; B*40:01; B*44:02; B*57:01. Immunogenicity of MHC-I epitopes was evaluated using IEDB’s class I immunogenicity tool (http://tools.iedb.org/immunogenicity/) that predicts immunogenicity of an epitope-MHC complex through the analysis of the location and properties of amino acids. A positive score indicates probable T-cell recognition, while a negative score suggests reduced likelihood (54).

MHC class II epitopes (predicted CD4+ T-cell epitopes) were identified using the IEDB Consensus tool (http://tools.iedb.org/mhcii/), employing a 7-allele reference panel: DRB1*03:01; DRB1*07:01; DRB1*15:01; DRB3*01:01; DRB3*02:02; DRB4*01:01; DRB5*01:01. Peptides with a percentile rank <0.5 and IC50 <50 nM (high affinity) or <500 nM (intermediate affinity) were retained (55).

Linear B-cell epitopes in exotoxins, Grp94, Grp78, and HSP70 were predicted using BepiPred-2.0 sequential B-cell epitope predictor (https://services.healthtech.dtu.dk) (56). A threshold score above the default 0.5 was used to increase specificity and reduce false positives.

## Results

### Sequence and structural similarity of streptococcal exotoxins

**Figure 2:**
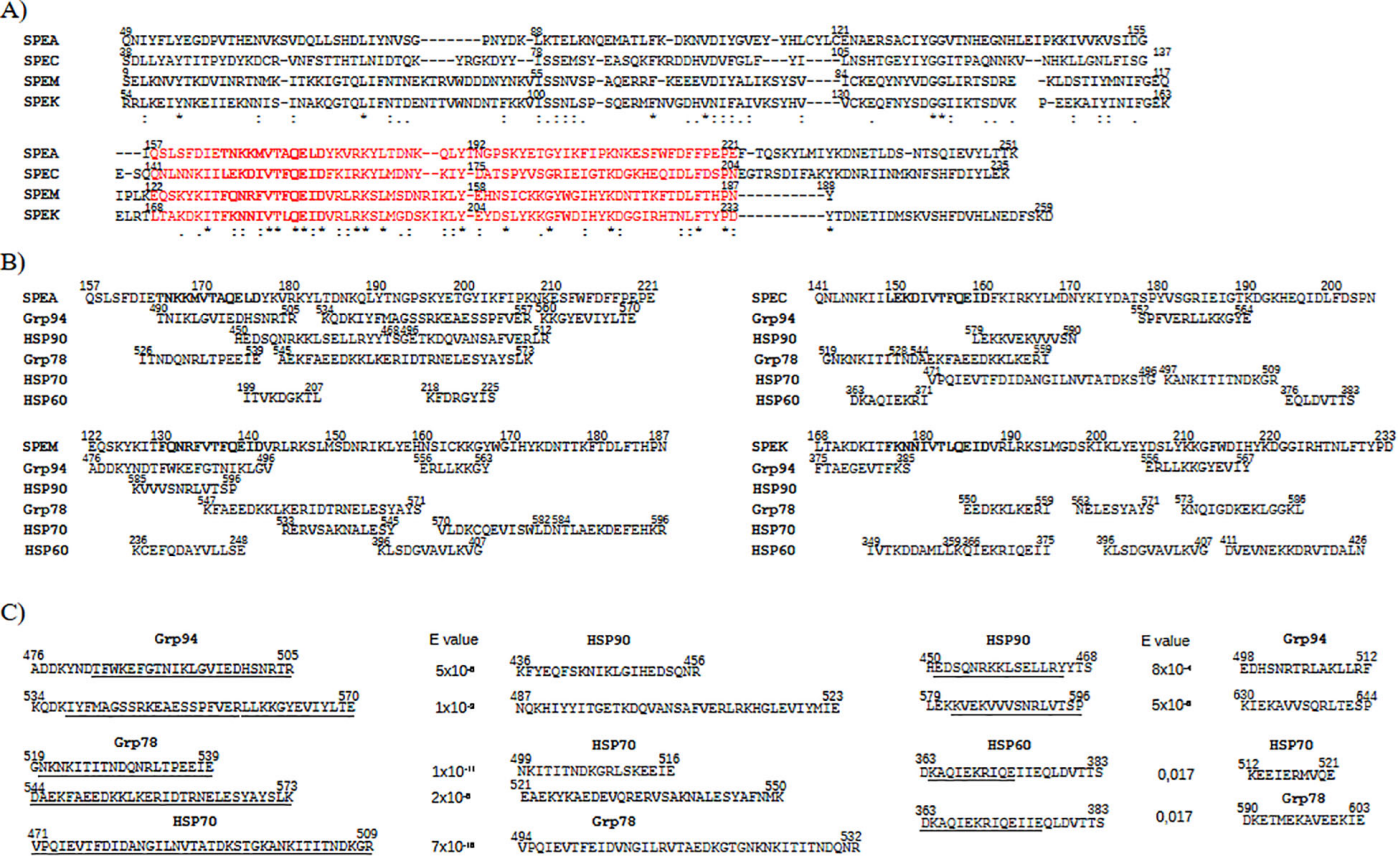
HSP sequences showing high similarity to the conserved superantigen (SAg) region of streptococcal exotoxins. **(A)** Multiple sequence alignment of exotoxins SPEA, SPEC, SPEM, and SPEK, showing sequence conservation in the SAg region: SPEA (aa 157–221), SPEC (141–204), SPEM (122–187), and SPEK (168–233) (highlighted in red). In bold the SAg domain sequence. Identical (*), conserved (): and semi-conserved (.) residues are indicated. **(B)** The HSP sequences are shown that aligned with the SAg regions of exotoxins in pairwise comparisons (see **Supplementary Figure S2**). **(C)** For each HSP sequence as in **(B)**, the homologous counterpart in other HSPs was searched in BLAST. The identified new HSP sequences are reported on the right of the original HSP sequences (underlined the corresponding similar sequence). All sequences displayed high similarity (low E-values) and aligned with exotoxins with high confidence (T-Coffee score = 1000).

Four major exotoxins of *Streptococcus pyogenes*, SPEA, SPEC, SPEM, and SPEK, were analyzed as representative members of the bacterial SAg toxin family. Despite differences in length, multiple sequence alignment using T-Coffee revealed high consensus scores across all toxins, reflecting conserved sequences (**Figure 2A**), consistent with previous observations in other bacterial SAgs (22). The region showing the greatest conservation spanned amino acids 157–221 of SPEA and the corresponding aligned segments in SPEC, SPEM, and SPEK, which included a 12-residue sequence (bold in **Figure 2A**) corresponding to the β-strand/hinge/α-helix domain, known to be highly conserved among *Staphylococcus aureus* and *Streptococcus pyogenes* SAgs (22). Similar alignment results were confirmed using Clustal Omega (data not shown).

Structural alignment using Expresso (T-Coffee) also revealed strong similarity among exotoxins, particularly within the SAg domain, in both pairwise (**Supplementary Figure S1A**) and multiple alignments (**Supplementary Figure S1B**). The overlap of sequence and structural homology in this domain (**Figure 2A** and **Supplementary Figure S1**) supports previous findings that conservation within this region underlies a shared mechanism of action across SAgs (22), indicating potential for similar biological activity (28, 57).

### Broad sequence similarity between streptococcal exotoxins and HSPs

Pairwise alignments of each exotoxin (SPEA, SPEC, SPEM, SPEK) with Grp94, HSP90, Grp78, HSP70, and HSP60 (**Supplementary Figure S2**) showed that ~90% of SPEA, SPEC, and SPEM sequences aligned with Grp94. SPEM showed the highest coverage with HSP90 (99%), Grp78 (91%), and HSP70 (92%). Each exotoxin sequence was largely covered by overlapping segments from at least two HSPs, especially for SPEA and SPEC (**Supplementary Figure S2A**). Long contiguous HSP segments, often over 30 amino acids, matched exotoxin regions with minimal gaps. To exclude chance similarity, exotoxins were aligned with seven unrelated human proteins (albumin, antitrypsin, beta2 microglobulin, apolipoprotein A, adiponectin, kallikrein, vitamin D-binding protein). In comparison with exotoxins, the control proteins consistently showed lower sequence identity (average ≤60%) and shorter aligned segments (data not shown).

### Specific HSP sequences match streptococcal exotoxins in the SAg domain

Given the immunological relevance of the SAg domain (22, 25, 27, 57), HSP sequences overlapping this region were examined (**Figure 2B**). Only HSP segments fully aligned with the SAg domain in pairwise alignments (**Supplementary Figure S2**) were included. Selection criteria were segment length and alignment with multiple exotoxins. Due to the greater length of HSPs relative to exotoxins, matches with common regions of multiple exotoxins supported sequence significance. Grp78 (residues 544–586) and Grp94 (534–570) overlapped various exotoxin segments, often with internal overlap. HSP70 (471–509) aligned with SPEC and partly overlapped its SAg domain. HSP90 and HSP60 showed only sparse, short matches in this region (**Figure 2B**).

Given the high sequence similarity between HSP family members (Grp94/HSP90: 92%; Grp78/HSP70: 96%), homologous segments of aligned HSP sequences were sought. Additional matching segments were thus identified (**Figure 2C**): Grp94 476–505 and 534–570 aligned, respectively, with HSP90 436–456 and 487–523; reciprocally, HSP90 450–468 and 579–596 matched, respectively, Grp94 498–512 and 630–646. HSP70 471–509 corresponded to Grp78 494–532, and Grp78 544–573 aligned with HSP70 521–550. Grp78 519–539 matched HSP70 499–516, while Grp78 573–586 lacked a homolog in HSP70, delimiting alignment to the residue 573. Combined, these homologous sequences in Grp78/HSP70 formed nearly continuous segments (HSP70: 471–550; Grp78: 494–573) separated by a five-residue gap. HSP60 displayed minimal sequence similarity to other HSPs (data not shown), with only two short regions (363–383) aligning with HSP70 (512–521) and Grp78 (590–603) (**Figure 2C**).

These additional homologous HSP sequences aligned with the same exotoxin segments as the originals. The results thus showed that Grp94, and especially Grp78 and HSP70, contained distinct segments with significant similarity to the exotoxin SAg region.

### Similarity of MHC-I epitopes between streptococcal exotoxins and HSPs

To assess whether the HSP sequences with significant similarity to the SAg region also exhibited immunogenic potential, we analyzed their content of MHC-I epitopes and compared them with those of streptococcal exotoxins. The necessary premise to this was that the exotoxins, like other foreign proteins, are internalized into antigen-presenting cells (APCs) and presented in complex with MHC-I and MHC-II molecules to TCRs (58). Only epitopes with high predicted binding affinity (IC50 <500 nM) were selected from exotoxins and HSPs (**Supplementary Tables S1**, **S2**).

Exotoxin epitopes were predominantly localized in the N-terminal and SAg regions. While isolated epitopes were observed in the N-terminus, a notable concentration of overlapping epitopes was found in the SAg domain: six in SPEA 173–204, six in SPEC 157–187, five in SPEK 182–211, and even thirteen in SPEM 125–188, despite SPEM being the shortest exotoxin (**Table 1**). All epitopes were allele-specific, except one epitope, each in SPEC and SPEM, which bound two distinct alleles.

**Table 1:** Sequences of both exotoxins and HSPs identified as MHC-I epitopes with the best binding affinity to HLA-A and HLA-B alleles.

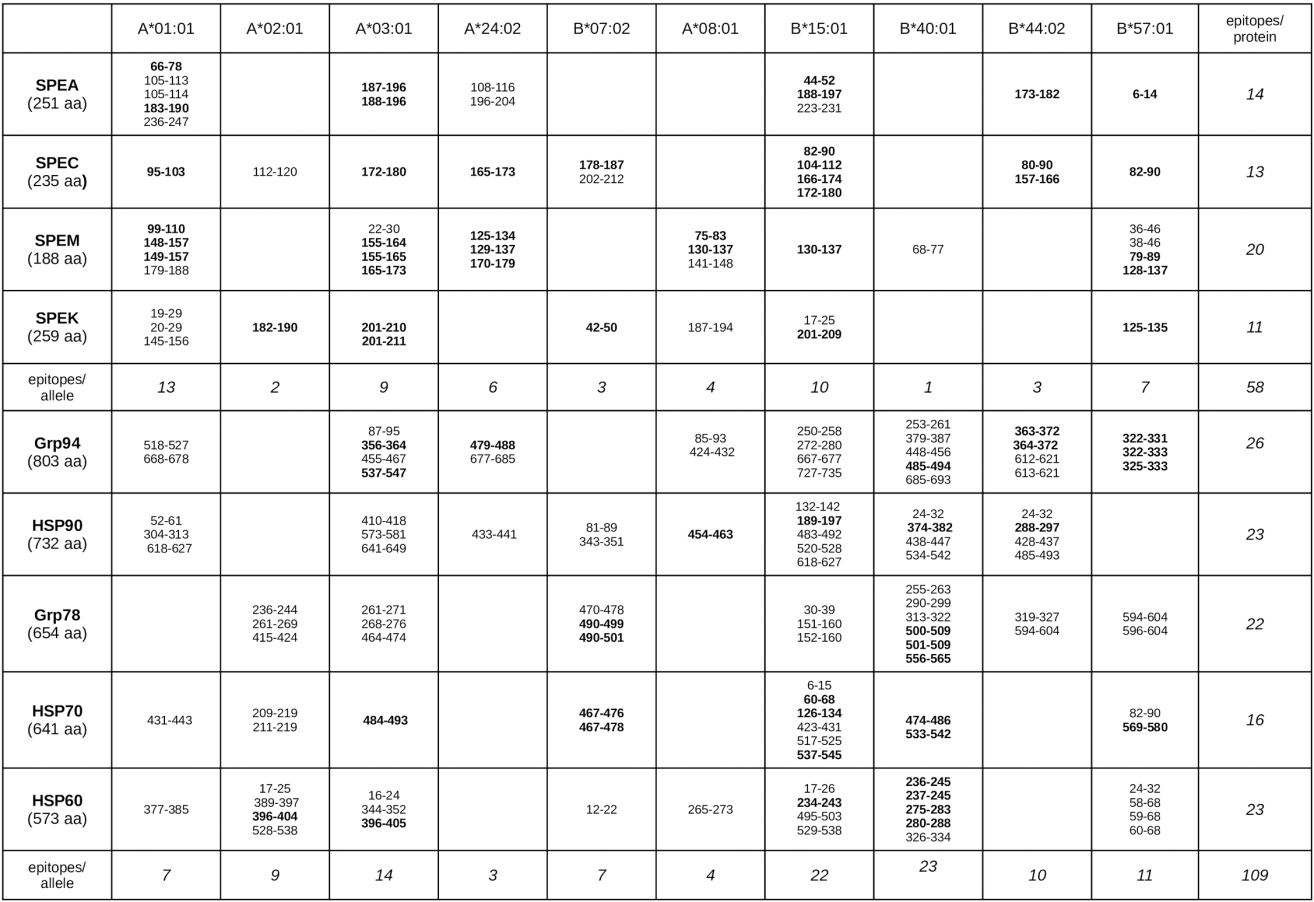

For each protein (length in parentheses), the table lists all MHC-I epitopes identified using IEDB tools in exotoxins (**Supplementary Table 1**) and HSPs (**Supplementary Table 2**) Each epitope is shown with the binding affinity to a specific HLA-A or HLA-B allele. Bolded sequences indicate matching epitopes between exotoxins and HSPs, as shown in pairwise alignments (**Supplementary Figures 2**, **3**). The right column lists the total number of epitopes per protein; the bottom row shows the total number of epitopes per allele group.

Among HSPs, Grp94, HSP90, Grp78, and HSP60 contained numerous epitopes, whereas HSP70 exhibited fewer total epitopes but all concentrated within the SAg-similar regions (**Table 1** and **Supplementary Table S2**). The most frequently targeted alleles included HLA-B40:01 and B15:01, the latter shared with several exotoxin epitopes. Multiple HSP sequences contained overlapping epitopes, particularly within Grp94, HSP90, Grp78, and HSP70 (**Table 1**).

**Figure 3:**
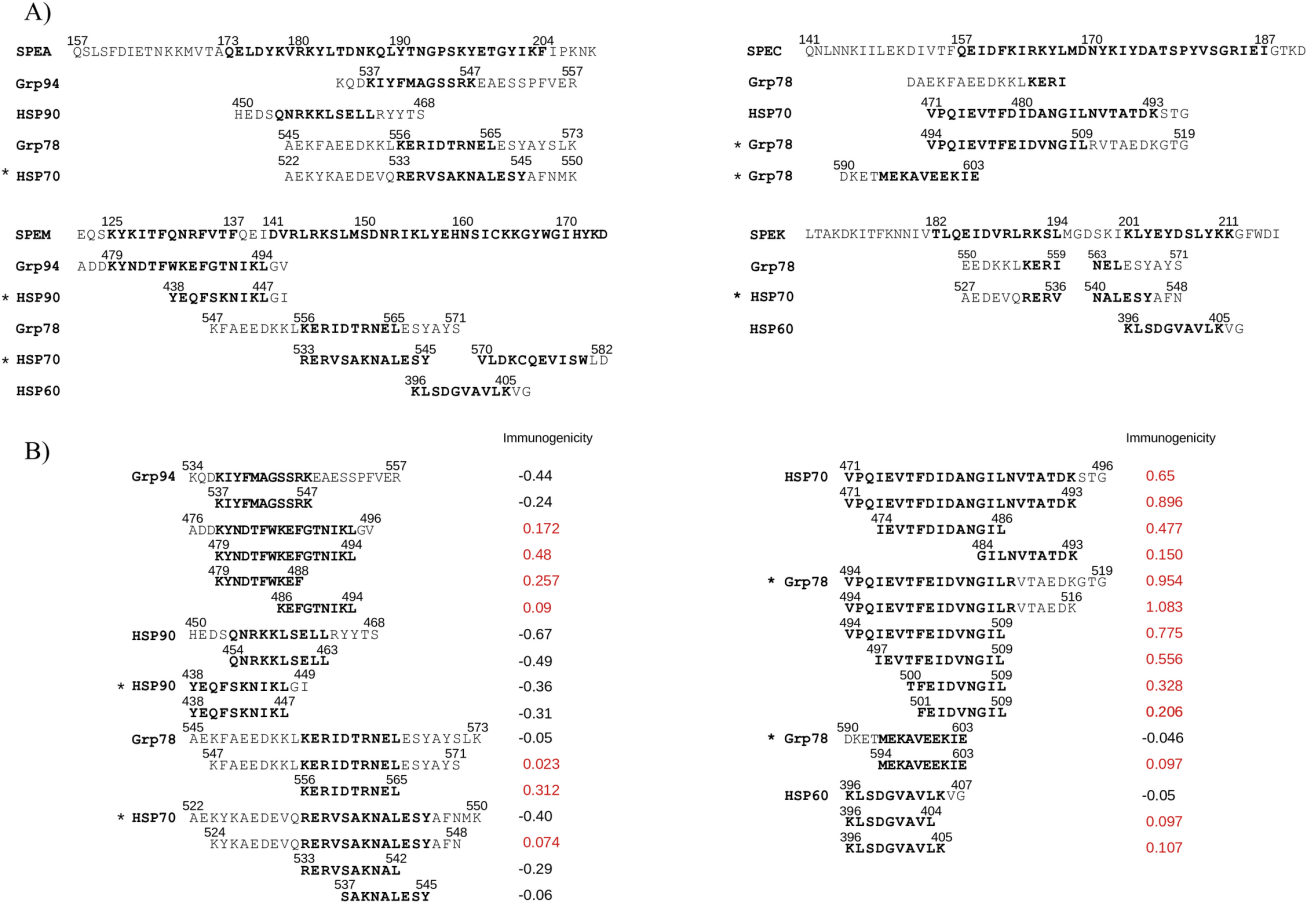
MHC-I epitope immunogenicity of HSPs matching exotoxin sequences in the SAg region. **(A)** HSP sequences aligned with exotoxins (from **Figure 2B**) that contain MHC-I epitopes (bold) overlapping those in the SAg region. Asterisks (*) indicate homologous HSP sequences derived from alignments in **Figure 2C**. **(B)** Class-I immunogenicity scores of the epitopes in **(A)**, with positive values (in red) indicating potential for T-cell recognition of the peptide -MHC complex.

To determine overlapping between HSP and exotoxin epitopes, MHC-I epitope mapping was performed along previously aligned regions (**Supplementary Figure S2**). Many HSP epitopes, especially in Grp94 and HSP70, closely matched those of SPEM and SPEA (**Supplementary Figure S3**). Notably, these epitopes were localized within the same sequences previously aligned to the SAg region, which itself harbored a dense cluster of overlapping epitopes (**Figure 3A**). In contrast, matching epitopes outside the SAg region were fewer and spatially isolated (**Supplementary Figure S3**), reinforcing the functional significance of the SAg domain in immune stimulation. Importantly, the extended sequences of Grp78 and HSP70, each forming a single long homologous stretch (**Figure 2C**), contained epitopes overlapping with those of all exotoxins, particularly SPEA, SPEC, and SPEM (**Figure 3A**).

While some sequences of Grp94, HSP90, and HSP60 aligned with the SAg domain, not all contained MHC-I epitopes. For instance, Grp94 476–496, aligned with SPEM, contained three epitopes, whereas the longer 534–570 segment, aligning with multiple exotoxins, included only one epitope in the sequence 534–557 aligned with SPEA (**Figure 3A**). HSP90 and HSP60 contributed fewer matching epitopes, two and one, respectively. Collectively, only the Grp78 and HSP70 sequences provided consistent, extensive overlap with the immunodominant SAg regions of all exotoxins.

### Immunogenicity of HSP sequences containing MHC-I epitopes

Beyond binding affinity, T-cell immunogenicity is a critical determinant of functional epitope recognition (54). We therefore assessed the predicted immunogenicity of each HSP epitope with sequence similarity to exotoxins. Only epitopes with positive immunogenicity scores were considered functionally relevant (**Figure 3B**), although all had high MHC-I binding affinity (**Supplementary Figure S3**).

Among all peptides analyzed, the HSP70 sequence 471–493 and its homologous Grp78 494–516 displayed the highest immunogenicity, containing distinct, strongly immunogenic epitopes (**Figure 3B**). The Grp78 547–571 and the homologous HSP70 524–548 were moderately immunogenic, while the Grp78 556–565 epitope was more potent. Grp94 476–496, with three overlapping epitopes, also showed positive immunogenicity, unlike the Grp94 534–570 segment and all epitopes within HSP90, which were non-immunogenic. HSP60 epitopes displayed weak but detectable immunogenicity (**Figure 3B**).

Thus, only select regions of Grp78 and HSP70, specifically those also aligning with the SAg domains of exotoxins, appeared to possess strong immunogenic responses, supporting their potential to functionally mimic bacterial SAgs.

### HSP70 and Grp78 sequences contain multiple epitope classes

While cytotoxic T lymphocytes are central to the anti-tumor immune response, both B cells and CD4^+^ T helper cells are essential for antigen recognition and the orchestration of adaptive immunity (59). To assess the immunogenic potential of HSP sequences homologous to the SAg domain (**Figure 3B**), the most predictive B-cell and MHC class II epitopes were identified within these regions. Exotoxins were analyzed in parallel for comparison.

**Figure 4:**
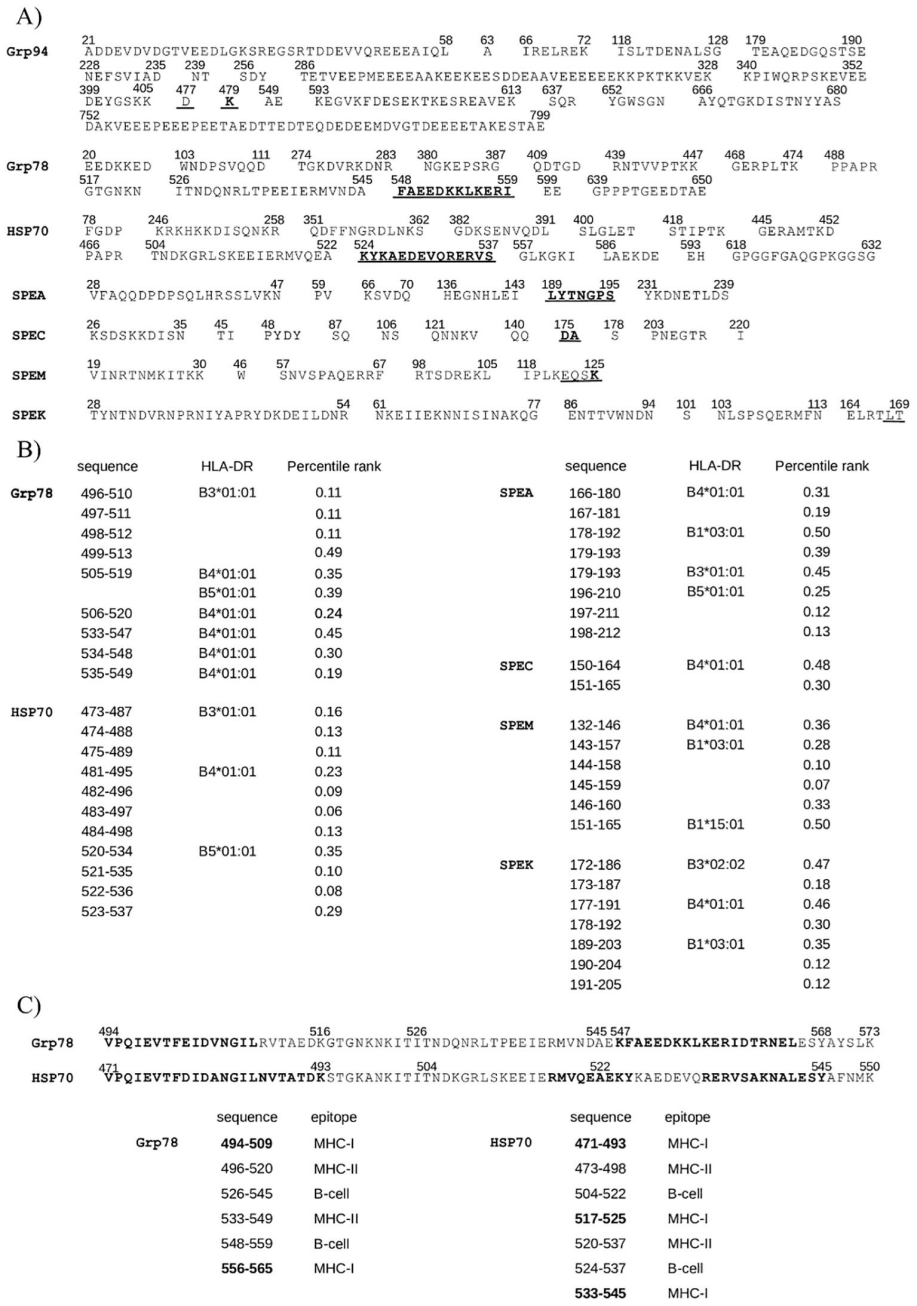
Grp78 and HSP70 sequences containing both MHC-I and B-cell epitopes also include MHC-II epitopes. **(A)** B-cell epitopes predicted by BepiPred-2.0 in HSPs and exotoxins. Underlined: B-cell epitopes with cross-similarity to the SAg region (**Figure 2B**); bold: epitopes also identified as MHC-I epitopes (**Table 1**). **(B)** Top-ranking MHC-II binding peptides in Grp78, HSP70, and exotoxins, with percentile rank (≤0.5) and binding allele listed. Shown are only those from cross-similar regions. Grp94 476 -496 did not yield any strong DRB-binding epitopes (**Supplementary Table S3**). **(C)** Immunogenic regions 494 -573 of Grp78 and 471 -550 of HSP70 showing class-I (bold), class-II (overlapping with class-I and B-cell epitopes on the left), and B-cell epitopes (middle/right).

Grp94 harbored several long B-cell epitopes, most of which did not overlap with its class I immunogenic sequences (**Figure 4A**). Only two residues, D477 and K479, located within the 476–496 region, served as both B-cell and class I epitopes. K479 (in bold) was the sole residue common to both (**Figure 3**). In contrast, the sequences 548–559 of Grp78 and its homologous region 524–537 in HSP70 functioned as B-cell epitopes, with partial overlap of class I epitopes (**Figure 4A**, underlined and bold), a result that supported the broad immunogenicity of the homologous sequences of Grp78 and HSP70. Compared to HSPs, exotoxins displayed a relatively limited number of B-cell epitopes, with overlapping B-cell and class I epitopes confined to short segments in SPEA, SPEC, and SPEM within the SAg domain.

A search for high-affinity MHC class II epitopes (percentile rank ≤0.50) across a representative panel of seven prevalent HLA class II alleles (**Figure 4B**) identified four Grp94 epitopes within residues 534–551, outside the class I immunogenic region (**Supplementary Table S3**). Notably, Grp78 and HSP70 contained nine and eleven class II epitopes, respectively. Of the Grp78 epitopes, six were found within residues 496–520, homologous to HSP70 473–498 (**Figure 2C**) and three within the sequence 533–549. Among the eleven HSP70 epitopes, seven mapped to residues 473–498 and four to 520–537, all within the immunogenic 471–550 segment shared with Grp78 544–573 (**Figure 2C**).

Exotoxins, particularly SPEA, SPEM, and SPEK, featured multiple class II epitopes overlapping class I epitopes in the SAg domain (see also **Figure 3A**). These epitopes predominantly bound to DRB1*03:01 (nine epitopes) and DRB4*01:01 (seven epitopes), the latter also targeted by epitopes in Grp78 and HSP70. Additional overlaps were found with DRB5*01:01 and DRB3*01:01, shared among both HSPs and exotoxins (**Figure 4B**). Grp78 (residues 505–549) and HSP70 (481–498) featured class II epitopes binding to DRB4*01:01, the same allele recognized by epitopes in the SAg regions of SPEA, SPEC, SPEM, and SPEK. Interestingly, the strongest binding affinity to DRB4*01:01 was observed for HSP70 epitopes within 482–497. Similarly, Grp78 and HSP70 epitopes targeting DRB3*01:01 demonstrated superior binding affinities compared to the corresponding SPEA epitope in the sequence 179–193 (**Figure 4B**).

The homologous 80-residue sequences of Grp78 and HSP70, formed by combining original and cross-matching segments (**Figure 2C**), contained overlapping epitopes of all three classes. Notably, regions Grp78 494–520 and HSP70 471–498 featured near-complete overlap of class I and class II epitopes (**Figure 4C**, bold). Additional overlapping B-cell and class II epitopes extended from 526 to 559 in Grp78 and from 504 to 537 in HSP70. Remarkably, within HSP70 517–527, epitopes of all three classes coincided. One particularly immunogenic segment was the B-cell epitope 526–545 in Grp78, which also included three consecutive class II epitopes. This region contained residues 526–539, homologous to SPEA 163–176, encompassing the SAg domain motif ^165^TNKKMVTAQELD^176^ (**Figure 2B**). This SPEA segment also included two class II epitopes with affinity for the same allele to which also the Grp78 epitopes bound (**Figure 4B**). Another significant region, HSP70 480–511, aligned with SPEM 105–136 in pairwise alignments (**Supplementary Figure S2**). This SPEM segment, which included the SAg motif ^130^FQNRFVT^136^ (**Figure 3A**), contained class I epitopes that overlapped two class I epitopes of HSP70 480–493 (**Supplementary Table S2**).

**Figure 5:**
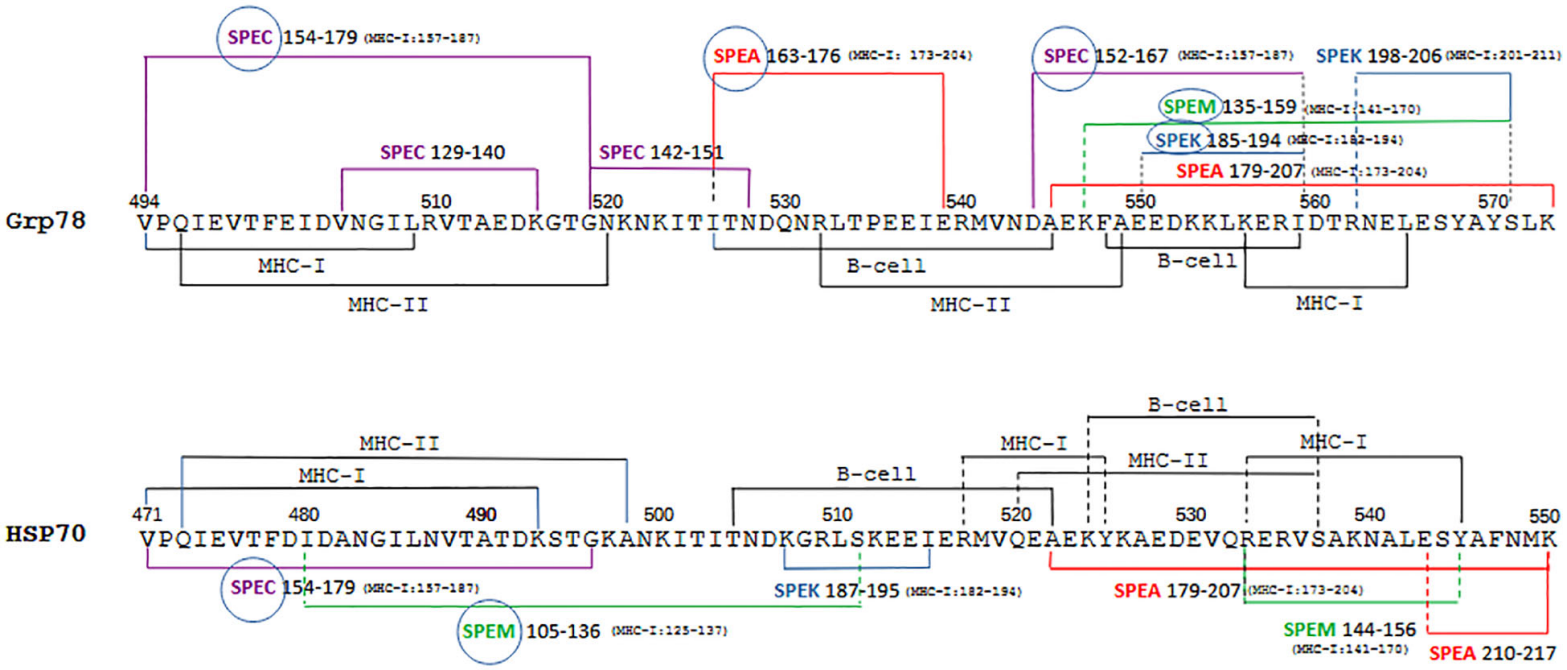
Summary visualization of epitopes of different classes and similar exotoxin sequences in the Grp78 and HSP70 sequences. The two homologous immunogenic sequences of Grp78 and HSP70 are shown with highlighted the regions covered by frequently overlapping MHC-I, MHC-II and B-cell epitopes, together with segments showing similarity to exotoxins. Where applicable, next to the exotoxin sequence is indicated in parenthesis the corresponding sequence range containing MHC-I epitopes (see **Figure 3A**). For sake of clarity, class II epitopes of exotoxins are not depicted. Encircled exotoxins represent those whose sequences partly or entirely contain the SAg domain (see **Figure 2B**).

**Figure 5** illustrates more clearly how the sequences of Grp78 and HSP70 are composed almost entirely of overlapping epitopes from different classes. While the left regions of both HSP sequences contain nearly identical types of epitopes of similar lengths, the right regions show some differences. Specifically, the HSP70 segment (residues 517–545) includes peptides that function as both MHC-II and B-cell epitopes and also overlap more extensively with MHC-I epitopes compared to the corresponding Grp78 segment (residues 540–568).

Similarly, the left regions of both HSP sequences exhibit similarity exclusively with specific sequences from SPEC and SPEM. In contrast, the right regions share similarities with a broader range of exotoxin sequences. Notably, it is the Grp78 sequence—in contrast to HSP70—that shows a more extensive resemblance to all exotoxin sequences, many of which contain multiple MHC-I epitopes.

A careful inspection of the multiple exotoxin sequences aligned with the HSP sequences also shows how broad is the overall similarity coverage by both Grp78 and HSP70 of SPEA (163–217), SPEC (129–179) and SPEM (105–159). This similarity fully encompasses the shared SAg domain of these exotoxins (see **Figure 2A**). Less extensive similarity is instead observed between the HSP sequences and SPEK (185–206), although its SAg region is partially covered, mostly by Grp78 (**Figure 5**).

### Homologous immunogenic sequences of HSP70 and Grp78 display significant similarity to tumor antigens 5T4 and CEA

The preceding findings supported the notion that the immunogenic sequences of Grp78 and HSP70 possess intrinsic capacity to trigger a complete immune response, comparable, or even superior, to that elicited by streptococcal SAg exotoxins. These sequences, when presented as tumor Ags, may therefore have the potential to activate effective anti-tumor immunity. Despite extensive experimental evidence demonstrating their role as tumor Ags, HSPs have not yet been translated into clinical use as diagnostic or prognostic biomarkers. To explore this possibility further, we examined whether the immunogenic regions of Grp78 and HSP70 exhibited sequence similarity with tumor-associated antigens currently used in clinical practice, namely 5T4 and CEA. The trophoblast glycoprotein 5T4 is a cell surface antigen overexpressed across a broad spectrum of malignancies, with limited expression in normal tissues (60). Although 5T4 has been evaluated as a target for tumor immunotherapy (18), clinical outcomes have remained modest (35, 36, 61).

**Figure 6:**
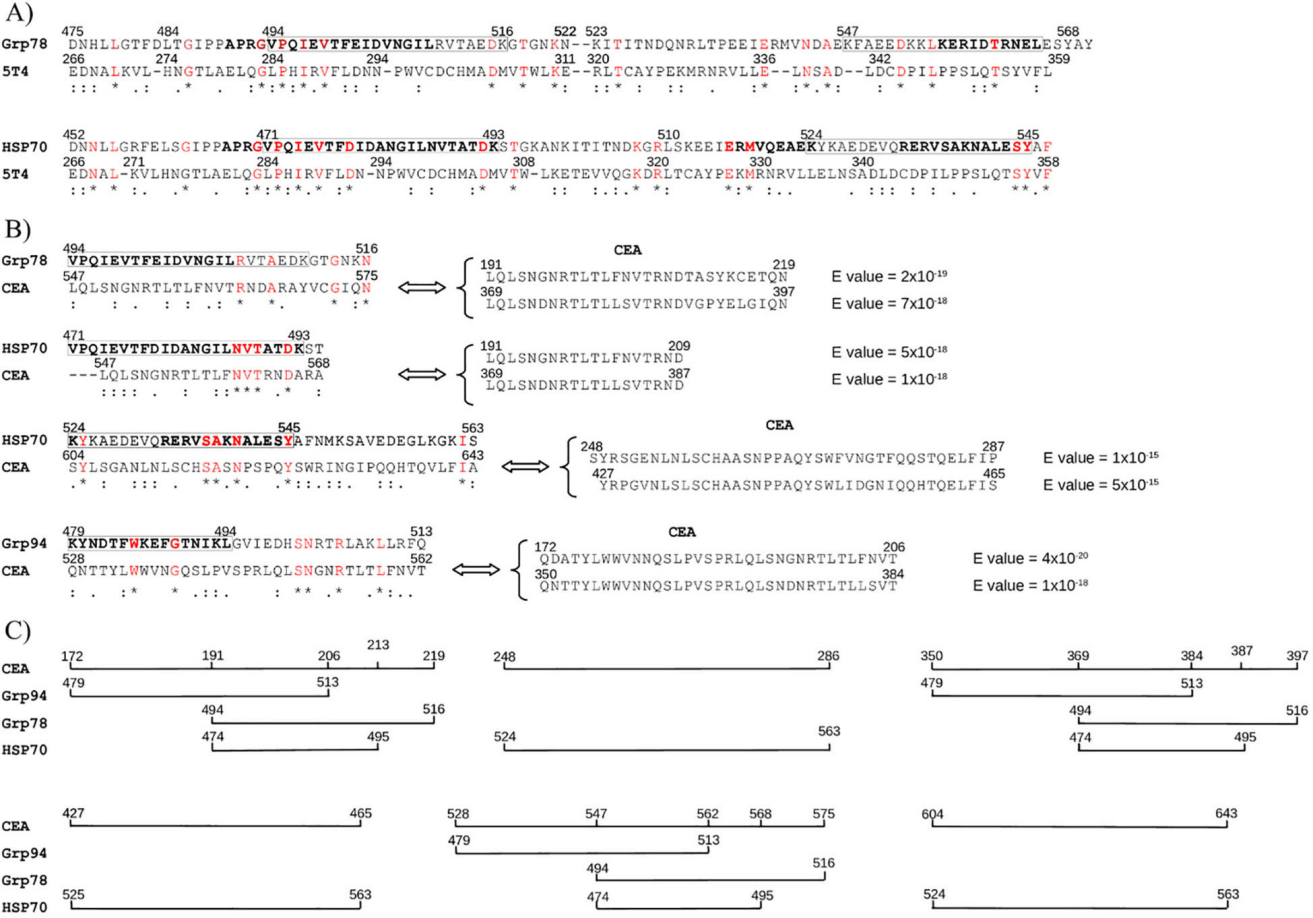
Sequence similarity between immunogenic Grp78/HSP70 regions and tumor markers 5T4 and CEA. **(A)** Pairwise alignments show 5T4 sequences with similarity to immunogenic regions of Grp78 and HSP70. Boxed regions correspond to class-I epitopes (bold, see **Figure 3B**). No significant similarity was found between 5T4 and Grp94. Below sequences: * (red) = identical; : = conserved; . = semi-conserved residues. **(B)** CEA sequences with similarity to Grp78 and HSP70 based on pairwise alignment. Each CEA sequence also showed strong similarity (BLAST, low E-values) to at least two other CEA regions, indicating intra-gene family homology. **(C)** Graphical representation of CEA sequence coverage by HSP-matching regions, showing the extent of continuous similarity to Grp78 and HSP70.

No sequence similarity was observed between 5T4 and the immunogenic region of Grp94 476–496. In contrast, both Grp78 and HSP70 exhibited high-scoring sequence alignments with 5T4. Pairwise alignments conducted using both T-Coffee and Clustal Omega revealed a high degree of similarity, with the strongest alignment occurring between residues 493–504 of Grp78 and 470–479 of HSP70 and the 283–292 region of 5T4 (**Figure 6A**). Multiple sequence alignment further confirmed this observation, yielding a consensus score exceeding 90% when the full-length sequences of Grp78 and HSP70 were aligned with 5T4 (**Supplementary Figure S4**), a result not replicated with any other HSP.

Next, sequence similarity was assessed with CEA, a well-characterized glycoprotein of the immunoglobulin superfamily and a widely used biomarker in various cancers (62). As with 5T4, Grp94 did not show any significant similarity to CEA. However, homologous immunogenic sequences of Grp78 and HSP70 did display notable alignment with CEA. Unlike 5T4, where the similarity was confined to a single segment, CEA exhibited multiple, discrete regions of similarity. This is consistent with the genomic architecture of the CEA family, which comprises a series of tandem gene duplications encoding repeated amino acid motifs (62).

Grp78 sequences 494–517 and 547–568, both within the broader 494–568 region, aligned with CEA sequences, with which also the HSP70 471–493 segment showed similarity (**Figure 6B**). Additionally, the HSP70 region spanning residues 524–545 aligned with three other distinct CEA sequences. When mapped sequentially, the regions of CEA showing similarity to HSP sequences revealed that HSP70 aligned with a broader set of CEA segments (**Figure 6C**). Notably, while the left portions of the HSP sequences (Grp78 493–504 and HSP70 470–479) were more similar to 5T4 (**Figure 6A**), their right portions (Grp78 547–568 and HSP70 524–545) demonstrated greater similarity to CEA (**Figure 6B**).

These findings reinforce the immunogenic relevance of the Grp78 and HSP70 sequences and support their potential involvement in tumor immunity through cross-reactivity with clinically recognized tumor antigens.

## Discussion

In this study, we tested the hypothesis that the antitumor effects observed in bacteria-based therapies could result from an adaptive immune response initiated by sequence similarity between bacterial SAgs and specific HSP sequences known to function as tumor Ags. Considering the significant impact that various streptococcal preparations have demonstrated in cancer therapy over time (10, 12, 21), we selected the streptococcal exotoxins SPEA, SPEC, SPEM, and SPEK as model SAgs to test this hypothesis.

It is well established that bacterial and viral SAgs share conserved sequence homology within the SAg domain, which is associated with a common structural conformation that underpins their potent immunostimulatory activity (22–24). Our analysis confirmed that the streptococcal exotoxins exhibit the highest degree of sequence and structural similarity around the SAg domain and its adjacent regions (**Supplementary Figure S1B** and **Figure 2A**), despite interindividual sequence variations.

The rationale for comparing HSPs to these bacterial exotoxins stems from the knowledge that HSPs exhibit extensive sequence conservation across species within their respective families and are recognized as immunodominant antigens in various bacterial and viral infections (63–65). Molecular mimicry between bacterial and human HSPs has been implicated in the pathogenesis of different inflammatory diseases due to antigenic cross-reactivity (66–68). Moreover, several HSPs, especially Grp94, HSP90, Grp78, HSP70, and HSP60 are involved in the development and progression of various tumors (40, 44, 46, 48, 49).

Pairwise alignment analyses revealed that not only there was an extensive similarity between HSPs and exotoxins (**Supplementary Figure S2**), not shared by any of the unrelated control proteins, but also that the highest density of similarity precisely occurred in the region of the SAg domain. Certain HSPs, specifically Grp94, Grp78, and HSP70, displayed extensive, crucial similarity that in the case of Grp94 and Grp78 involved the SAg region of all tested exotoxins with segments of a single, uninterrupted sequence. This finding was of relevance in consideration of the different length of the HSPs compared to that of exotoxins. Although HSP70 was found to align exclusively with SPEC, the alignment was considered significant in that it occurred with an uninterrupted long stretch of HSP70 that nearly covered the entire SAg region (**Figure 2B**).

Given the inherent sequence homology among HSPs of the same class, we anticipated that additional, previously unidentified homologous sequences would exist that exhibit similar degrees of similarity to the exotoxins. Indeed, homologous sequences were found between HSP70 and Grp78, as well as between Grp94 and HSP90, whereas only short homologous segments were identified between HSP60 and either HSP70 or Grp78 (**Figure 2C**). These findings revealed that Grp78 and HSP70, when considered together with their homologous counterparts, formed a nearly continuous 80-amino acid-long sequence, a feature not observed with Grp94 or HSP90, thereby underscoring their potential functional importance.

To assess whether the HSP sequences aligned with the SAg domains possessed immunogenic properties comparable to those of exotoxins, we analyzed predicted MHC class I and II epitopes, as well as B-cell epitopes, within both HSPs and exotoxins. The results showed that MHC-I epitopes in the exotoxins were predominantly located within the SAg domain (**Supplementary Table S1**, **Supplementary Figure S3**), highlighting the immunogenic nature of this region and supporting its proposed internalization and presentation by APCs in complex with MHC class I molecules (15, 58, 69, 70).

Among the HSPs, only Grp78 and HSP70, and to a lesser extent Grp94, contained class I epitopes within the aligned sequences. Notably, the original and homologous sequences of Grp78 and HSP70 included numerous high-affinity MHC-I epitopes overlapping those of all exotoxins (**Figures 3A**). In particular, the original HSP70 sequence aligned with SPEC and its homologous in Grp78 contained peptides that also exhibited high predicted immunogenicity scores (**Figures 3A, B**).

Further analysis of MHC class II and B-cell epitopes reinforced the conclusion that Grp78 and HSP70 possess the highest immunogenic potential. In contrast to Grp94, these HSPs not only contained strong class I epitopes but also presented extensive class II and B-cell epitopes, suggesting their capacity to elicit robust humoral and cellular immune responses (**Figures 4**, **5**). The 80-amino acid sequences derived from the original and homologous segments of Grp78 and HSP70 were composed entirely of immunogenic regions: class I and II epitopes were predominantly located in the N-terminal regions (coinciding with the segments aligned to SPEC), while B-cell epitopes were localized toward the central and C-terminal portions (**Figure 4C**). These B-cell epitopes overlapped or laid adjacent to class II epitopes, forming a structurally and functionally integrated immunogenic unit.

Importantly, these epitopes not only exhibited high individual immunogenicity but also demonstrated extensive sequence similarity with the epitopes of the exotoxins. Furthermore, many of the class II epitopes from Grp78 and HSP70 shared high-affinity binding to the same HLA alleles as those from the exotoxins (**Figure 4B**), indicating functional mimicry at the level of antigen presentation.

The discovery that the immunodominant peptides within Grp78 and HSP70 align with the SAg domain of exotoxins and mimic their potent immunostimulatory properties has important implications. These HSP-derived sequences appear to function as endogenous analogs of bacterial SAgs, supporting the concept that human HSPs can exhibit antigenic cross-similarity with microbial proteins (38, 63–65). Within the context of streptococcal antitumor therapy, it is plausible that SAgs exert their effects by priming CD8+ T lymphocytes against HSP-derived epitopes that are overexpressed in tumor cells, either on the cell surface or as circulating soluble antigens.

This mechanistic link provides a basis for proposing that the HSP sequences identified here could function as tumor-specific antigens. Although various HSPs are well known for their roles in cancer biology and have been identified as tumor Ags (39, 40, 45–47, 49), none have yet been adopted as clinically validated tumor biomarkers. Our observation that the well-established tumor markers 5T4 and CEA exhibit substantial sequence similarity with the immunogenic regions of Grp78 and HSP70 (**Figure 6**) provides compelling support for the antigenic nature of these HSP sequences. This similarity likely reflects altered protein processing and expression accompanying oncogenic transformation (39). The upregulation of HSP genes in response to cellular stress (41, 42), particularly HSP70 and Grp94, is known to facilitate tumor antigen processing and presentation (39, 71–74). The fusion or stable association of HSPs with tumor-specific proteins may account for the observed high similarity between HSPs and tumor markers such as 5T4 and CEA. In addition to the global alignment similarity of HSP70 and Grp78 with 5T4 (**Supplementary Figure S4**), a result that as such deserves further investigation, it is particularly notable that immunogenic regions within Grp78 and HSP70 correspond closely to class I epitopes in 5T4 (data not shown) and also sustain a broader similarity with CEA (**Figure 6B**).

In summary, our in-depth bioinformatic investigation identified two homologous Grp78 and HSP70 sequences that closely mimic immunodominant epitopes of streptococcal SAg exotoxins. The additional finding of the strong similarity shared with two clinically relevant tumor antigens suggests that these HSP sequences may function as shared tumor antigens capable of eliciting a potent immune response, similar to that triggered by SAg exotoxins. Although these *in silico* findings have broad implications for tumor immunology—particularly in the development of vaccines and tumor biomarkers—caution is warranted in interpreting them as having immediate translational relevance. A key limitation of our study is the absence of *in vitro* or *in vivo* experiments needed to validate any bioinformatic predictions.

Although HSPs are obligatory intracellular proteins and therefore ignored by the immune system, their occasional surface expression or molecular mimicry with extracellular components cannot be ruled out, particularly following the administration of exogenous HSPs. Therefore, in addition to immunizing a host to assess the specific immune response to these HSP sequences, future research should also consider the potential risks of autoimmune reactions associated with administering highly immunogenic HSPs.

## Data Availability

The datasets presented in this study can be found in online repositories. The names of the repository/repositories and accession number(s) can be found in the article/**Supplementary Material**.

## Supplementary material

Supplementary Figure 1 Structure alignment of exotoxins. Exotoxins were structurally aligned using Expresso T-Coffee. **(A)** Pairwise alignments highlight the most similar SAg domain regions (in bold), each with a consensus score of 1000. **(B)** Multiple alignment starting from residue 42 of SPEA shows corresponding regions in SPEC, SPEM, and SPEK, with a consensus score of 890. In both **(A)** and **(B)**, symbols indicate identical (*), conserved (:), and semi-conserved (.) residues.

Supplementary Figure 2 Sequence alignment of exotoxins with HSPs. Pairwise alignments (see Methods) show only gap-free, similar sequences of ≥8 amino acids. SAg domain sequences are in bold.

Supplementary Figure 3 Predicted MHC-I binding peptides of HSPs matching exotoxin epitopes. MHC-I epitopes of HSPs (supplemental Table 2) overlapping exotoxin epitopes (supplemental Table 1) are shown based on alignments (supplemental Figure 2). Bold amino acids represent MHC-I peptides; non-bold are adjacent residues. Epitope numbers refer to alignment positions; bold residues adjacent to numbered epitopes refer to epitope residues that may or may not match known exotoxin or HSP epitopes. Multiple epitopes often exist within a single MHC-I peptide.

Supplementary Figure 4 Alignment of Grp78, HSP70, and 5T4. T-Coffee alignment highlights the regions with higher sequence consensus, excluding the first 73 and last 4 amino acids of 5T4 due to low similarity. An overall similarity score of 945 was found among the three proteins, with HSP70 showing the highest sequence consensus (960). Symbols indicate identical (*, red), conserved (:), semi-conserved (.) residues. Bold indicates shared MHC-I epitopes in Grp78 and HSP70 (**Table 1**).

Supplementary Table 1 Predicted best MHC-I peptides in exotoxins. Exotoxins were scanned for MHC-I peptides using IEDB (see Methods) with a set of common HLA–A, HLA–B alleles. IC50 values <500 nM (in parentheses) was considered for good binding.

Supplementary Table 2 Predicted best MHC-I peptides in HSPs. HSPs were scanned for MHC-I peptides using IEDB (see Methods) with a set of common HLA–A, HLA–B alleles. IC50 <500 nM (in parentheses) was considered for good binding.

Supplementary Table 3 Predicted best MHC-II peptides in exotoxins and HSPs. MHC-II peptides in exotoxins and Grp94, Grp78, HSP70 were predicted using IEDB (2023.05 method) with seven prevalent HLA alleles. Only top binders (percentile rank ≤0.5) were included.
