# Supplementary material for: Two homologous sequences of Grp78 and HSP70 represent tumor antigens shared with streptococcal superantigens in eliciting an antitumor immune response: an immunoinformatic investigation
Source: Front Immunol. 2025 Sep 11;16:1644687. doi: 10.3389/fimmu.2025.1644687 (PMC12460249; doi:10.3389/fimmu.2025.1644687)
Supplement: Supplementary Figure 1 — Structure alignment of exotoxins. Exotoxins were structurally aligned using Expresso T-Coffee. (A) Pairwise alignments highlight the most similar SAg domain regions (in bold), each with a consensus score of 1000. (B) Multiple alignment starting from residue 42 of SPEA shows corresponding regions in SPEC, SPEM, and SPEK, with a consensus score of 890. In both (A) and (B), symbols indicate identical (*), conserved (:), and semi-conserved (.) residues. [file DataSheet1.pdf]

Supplementary Figure S1

A)

```
       159                                183                                           217        226
SPEA   LSFDIETNKKMVTAQELDYKVRKYL--TDNKQLYTNGPSKYETGYIKFIPKNKESFWFDFF-PEPEFTQSK
       143                                   174                                                   210
SPEC   LNNKIILEKDIVTFQEIDFKIRKYLMDNYKI---YDATSPYVSGRIEIGTKDGKHEQIDLFDSPNEGTRSD
       *.  .*   :*.:** **:*:*:****   . :      :..* * :* *:: .*: :    :*:*     * *:*
```

```
       157                                   187                                     220
SPEA   QSLSFDIETNKKMVTAQELDYKVRKYLTDNK--QLYTNGPSKYETGYIKFIPKNKESFWFDFFPEP
       122                                          158                              186
SPEM   EQSKYKITFQNRFVTFQEIDVRLRKSLMSDNRIKLYE-HNSICKKGYWGIHYKDNTTKFTDLFTHP
       :.  .:.*   ::::** **:* ::** * .::   :**     *   :.**   :   *:: : : *:*..*
```

```
       158                                 188                    208
SPEA   SLSFDIETNKKMVTAQELDYKVRKYLTDNKQ--LYTNGPSKYETGYIKFIPKN
       169                                       204             220
SPEK   TAKDKITFKNNIVTLQEIDVRLRKSLMGDSKIKLYE-YDSLYKKGFWDIHYKD
       :  .  .*   ::::** **:* ::** * .:.:   **     * *:.*: .:   *:
```

```
       144                          167                                        204
SPEC   NNKIILEKDIVTFQEIDFKIRKYL--MDNYKIYDATSPYVSGRIEIGTKDGKHEQIDLFDSPN
       125                                                                     187
SPEM   KYKITFQNRFVTFQEIDVRLRKSLMSDNRIKLYEHNSICKKGYWGIHYKDNTTKFTDLFTHPN
       : ** ::: :*******.::** *    :. *:*: .*     *    *   **.. :   ***  **
```

```
       143                            167                                  200
SPEC   LNNKIILEKDIVTFQEIDFKIRKYL--MDNYKIYDATSPYVSGRIEIGTKDGKHEQIDLF
       170                                                                 229
SPEK   AKDKITFKNNIVTLQEIDVRLRKSLMGDSKIKLYEYDSLYKKGFWDIHYKDGGIRHTNLF
        ::** ::::***:****.::** *    .: *:*:   * * .*   :*   ***   .: :**
```

```
       124                                                                     187
SPEM   SKYKITFQNRFVTFQEIDVRLRKSLMSDNRIKLYEHNSICKKGYWGIHYKDNTTKFTDLFTHPN
       170                                                                     233
SPEK   AKDKITFKNNIVTLQEIDVRLRKSLMGDSKIKLYEYDSLYKKGFWDIHYKDGGIRHTNLFTYPD
       :* ****:*.:**:************.*.:*****::*: ***:*.*****.  :.*:***:*:
```

B)

```
       42                                                     79                                                                       120                                       154
SPEA   SSLVKNLQNIYFLYEGDPVTHENVKSVDQLLSHDLIYN--------VSGPNYDKLKTELKNQEMATLFKDKNVDIYGVEYYHLCYLCENAERSACIYGGVTNHEGNHLEIPKKIVVKVSID---
       31                                        60           68                                        107                                       126          136
SPEC   KDISNVKSDLLYAYTITPYDYKDCRVNFST-THTLNID------TQKYRGKDYYISSEMSYEASQKFKRDDHVDVFGLFYILNS------HTGEYIYGGITPAQNNKVN--HKLLGNLFIS---
       2                                         31                                      71           83                            106
SPEM   SDAVLVNSELKNVYTKDVINRTNMKITKKI-GTQLIFNTNRKTRVWDDDNYNKVISSNVSPAQERRFKEEE-VDIYALIKSYSV---ICKEQYNYVDGGLIRTSDREKLD-STIYMNIFGEQIP
       47                                        76                                                    129                            152         163
SPEK   KDEILDNRRLKEIYNKEIIEKNNISINAKQ-GTQLIFNTDENTTVWNDNTFKKVISSNLSPSQERMFNVGDHVNIFAIVKSYHV---VCKEQFNYSDGGIIKTSDVKPEE-KAIYINIFGEK-E
       ..        :   *          :     .     *  :                       :.:::.      :   . *::::.:              .       **:    .. .      :  ::  .
```

```
         157          165                     184                                          221                           243
SPEA   GIQS--LSFDIETNKKMVTAQELDYKVRKYL--TDNKQ--LYTNGPSKYETGYIKFIPKNKESFWFDFFPE-PEFTQSKYLMIYKDNETLDSNTSQ-IEVYLTTK
            141      149                    172                                     204                                235
SPEC   GESQQNLNNKIILEKDIVTFQEIDFKIRKYLMDNYK----IY-DATSPYVSGRIEIGTKDGKHEQIDLFDSPNEGTRSDIFAKYKDNRIINMKNFSHFDIYLEK-
        122          130                     149          159                     188
SPEM   LKE--QSKYKITFQNRFVTFQEIDVRLRKSL--MSDNRIKLYE-HNSICKKGYWQIHYKDNTTKFTDLFTHPNY-------------------------------
         167         176                     195          205                     233                          259
SPEK   LRT-LTAKDKITFKNNIVTLQEIDVRLRKSL--MGDSKIKLYE-YDSLYKKGFWDIHYKDGGIRHTNLFTYPDYTDNETIDMSKVSHFDVHLNEDFSKD------
                 . .*  :: :** **:* ::** *     .     :*     *    .*    :   *:        ::*
```
