# Supplementary material for: Two homologous sequences of Grp78 and HSP70 represent tumor antigens shared with streptococcal superantigens in eliciting an antitumor immune response: an immunoinformatic investigation
Source: Front Immunol. 2025 Sep 11;16:1644687. doi: 10.3389/fimmu.2025.1644687 (PMC12460249; doi:10.3389/fimmu.2025.1644687)
Supplement: Supplementary Figure 2 — Sequence alignment of exotoxins with HSPs. Pairwise alignments (see Methods) show only gap-free, similar sequences of ≥8 amino acids. SAg domain sequences are in bold. [file DataSheet2.pdf]

Supplementary Figure S2

1 10 20 30 40 50 60 70 80 90 100 110 120 130
SPEA MENNKKVLKKMVFFVLVTFLGLTISQEVFAQQDPDPSQLHRSSLVK**NLQNIYFLYEGDPV**THENVKSVD**QLLSHDLIYNVSGPNYDKLKTELKN**QEMATLFKDKNVDIYGVEYYHL**CYLCENAERSACIY**GG

105 134 154 166 204 215 324 365
Grp94 ISNASDALDKIRLISLTDENALSGNEELTV MTREELVKNLGT LVADKVIVTSKH KKVEKTVWDWELMNDIKPIWQRPSKEVEEDEYKAFYKSFSKE

98 110 291 319 364 376
HSP90 MTKADLINNLGTI NKTKPIWTRNPDDITNEEYGEFYKSLTND YVRRVFIMDNCEE

24 40 160 171 221 231 373 382 408 427
Grp78 KEDVGTVVGIDLGTTYS YLGKKVTHAVVT LVFDLGGGTFD QLVKEFFNGKDQDTGDLVLLDVCPLTLGIE

61 82 121 141 196 206 262 275
HSP70 LNPQNTVFDAKRLIGRKFGDPV SMVLTKMKEIAEAYLGYPVTN LIFDLGGGTFD RLRTACERAERSAC

1 11 14 66 91 106 149 159 181
HSP60 MLRLPTVFRQMVSRVLAPHLTRAYAKDVKFGADARALMLQGVDLLADAVAVTMGPKGRTVIIEQ KNIGAKLVQDVANNTN DAVIAELKKQS EIGNIISDAMKKVGRKGV

140 150 160 170 180 190 200 210 220 230 240 251
SPEA VTNHEGNHLEIPKKIVVKVSIDGIQSLSFDIE**TNKKMVTAQELDYKVRKYLTDNKQLYTNGPSKYETGYIKFI**PKNKESFWFDFFPEPEF**TQSKYLMIY**KDNETLDSNTSQIEVYLTTK

450 478 490 505 534 557 560 570 581 599
Grp94 TLQQHKLLKVIRKKLVRKTLDMIKKIADD TNIKLGVIEDHSNRTR KQDKIYFMAGSSRKEAESSPFVER KKGYEVIYLTEPEFDGKRFQNVAKEGVKFD

416 437 450 468 496 512 631 641
HSP90 LVKKCLELFTELAEDKENYKKF HEDSQNRKKLSELLRYYTSGETKDQVANSAFVERLR KKHLEINPDHS

437 446 526 539 545 573 578 596 605 623
Grp78 IPRNTVVPTK ITNDQNRLTPEEIE AEKFAEEDKKLKERIDTRNELESYAYSLKDKEKLGGKLSSEDKETMEK LESHQDADIEDFKAKKKEL

338 345 388 412 543 562
HSP70 GGSTRIPK VQDLLLLDVAPLSLGLETAGGVMTA ESYAFNMKSAVEDEGLKGKI

207 173 193 199 207 218 225 249 263
HSP60 ITVKDGKTL TISANGDKEIGNIISDAMKKV ITVKDGKTL KFDRGYIS KKISSIQSIVPALEI

10 20 30 40 50 60 70 80 90 100 110 120 130
SPEC MKKINIIKIVFIITVILISTISPIIKSDSKKDIS**NVKSDLLYAYTITPY**DYKDCRVN**FSTTHTLNIDTQKYRGKDYYISSEMSY**EASQKFKRDDHVDVFGLFYI**LNSHTGEYIY**GGITPAQNNKVNHKLLG

136 149 213 230 243 255 351 367
Grp94 IKCDKEKNLLHVTD SKHNNDTQHIWESDSNEF RGTTITLVLKEEA EEDEYKAFYKSFSKESD

41 49 103 122 215 231 275 288
HSP90 KEIFLRELILINNLGTIAKSGTKAFMEAL GYPITLFVEKERDKEVS KKKKKIKEKYIDQE

1 32 34 42 45 52 185 201 480 498 505
Grp78 MKLSLVAAMLLLLSAARAEEEDKKEDVGTVVGDLGTTYSCV FKNGRVEI KDAGTIAGLNVMRIINE GTFDLTGIPPAPRGVPQIEVNG

42 55 245 260 277 285 286 296 183 200
HSP70 VAFTDTERLIGDAA FKRKHKKDISQNKRAV STQASLEID SLFEGIDFYTS YGLDRTGKGERNVLIFDL

136 153 174 190 199 220 274 286 329 342
HSP60 NPVEIRRGVMLAVDAVIA ISANGDKEIGNIISDAM ITVKDGKTLNDELEIIEGMKFD IAEDVDGEALSTL GLTLNLEDVQPHDL

140 150 160 170 180 190 200 210 220 230
SPEC NLFISGESQQNLNNKII**LEKDIVTFQEIDFKIRKYLMDNYKIYDATSPYVSGRIEIG**TKDGKHEQIDLFDSPNEG**TRSDIFAKY**KDNRIINMKNFSHFDIYLEK

416 446 265 274 552 564 592 624
Grp94 VFITDDFHDMMPKYLNFVKGVVDSDDLPLNV KNLVKKYSQF SPFVERLLKKGYE AKEGVKFDESEKTKESREAVEKEFEPLLNWMK
D

493 510 579 590
HSP90 YITGETKDQVANSAFVER LEKKVEKVVVSN

516 519 528 544 559 567 577 599 626
Grp78 ILRVTAEDK GNKNKITITNDAEKFAEEDKKLKERI SYAYSLKNQIG EEKIEWLESHQDADIEDFKAKKKELEEI

379 395 471 496 497 509 520 539 582 601
HSP70 ILMGDKSENVQDLLLLD VPQIEVTFDIDANGILNVTATDKSTG KANKITITNDKGR QEARKYKAEDEVQRERVSAK DANTLAEKDEFEHKRKELEQ

363 371 376 383 405 429
HSP60 DKAQIEKRI EQLDVTTS KVGGTSDVEVNEKKDRVTDALNATR

SPEM 1 FSDAV **LVNSELKNVYTKDVI** NRTNMKIT **KKIGTQLIFNTNEKTRVWDDDNYNKVISSNVSP** AQERRFKEEEVDIYALIKSYSV **ICKEQYNYVD** GGLIRTSDREKLDSTIYMNIFGEQIPLKEQSKYKIT **FQ** 130
(position marks: 10, 20, 30, 40, 50, 60, 70, 80, 90, 100, 110, 120, 130)

Grp94 89 IINSLYKN 96 | 168 KSGTSEFLNNNDTQHIWESDS 176 216 227 | 337 NDIKPIWQRPSKEVEEDEYAFYKSFSKESDDPMAYIH 355 357 374 | 476 ADDKYNDTFW

HSP90 79 NLIPNKQDR 87 | 278 KKIKEKYIDQEELNKTKP 296 | 453 SQNRKKLSELL 463 | 585 KVVVS

Grp78 29 TVVGIDLGTTYSEIIANDQGNRIT 40 51 62 | 101 RTWNDPSVQQ 110 | 135 GQTKTFAPEEISAMVLTK 152 (I above K)

HSP70 1 MAKAAAIGIDLGTTYSNDQGNRTT 16 31 38 | 437 GVLIQVYEGERIDANGILNVTATDKSTGKANKITITND 447 480

HSP60 17 VLAPHLTRAYAKDV 30 | 92 NIGAKLVQDV 101 | 131 ISKGANPVEIRR 142 | 197 GVITVKDGKTLN 208 | 236 KCEFQ

SPEM **NRFVTFQEIDVRLRKSLMSDNRIKLYEHNSICKKGYWGIH** YKDNTTKFTDLFTHPNY
(position marks: 140, 150, 160, 170, 180)

Grp94 KEFGTNIKLGV 496 | 556 ERLLKKGY 563

HSP90 NRLVTSP 596

Grp78 547 KFAEEDKKLKERIDTRNELESYAYS 571

HSP70 KGRLS 511 | 533 RERVSAKNALESY 545 | 570 VLDKCQEVISWLDNTLAEKDEFEHKR 582 584 596

HSP60 DAYVLLSE 248 | 396 KLSDGVAVLKVG 407

SPEK MKKNTLTLLFLVCVSLALYTTESVFSDTYNTNDVRNPRNIYAPRYDKDEI **LDNRRLKEIYNKEII** EKNNISIN **AKQGTQLIFNTDENTTVWNDNTFKKVISSNLSP** SQERMFNVGDHVNIFAIVKSYHV **VC**
(position marks: 10, 20, 30, 40, 50, 60, 70, 80, 90, 100, 110, 120, 130)

Grp94 3 ALWVLGLCCVLLTF 16 | 54 EAIQLDGLNASQIRELRE 71 | 95 KNKEIFLRELISN 107 | 134 VKIKCDKEKNLLHVTDTGVGMTREELVKNLGT 165

HSP90 33 IINTFYSNKEIF 44 | 182 RGTKVILHLKEDQTEYLEERRIKEIVKKH 210 | 246 EKEEKESEDK 255 | 317 TNDWEDHLAVKH 328 | 350 DLFENRKKKNNIKLYVRRVFIMDNCEELI

Grp78 1 MKLSLVAAMLLLL 13 | 39 YSCVGVFKN 47

HSP70 42 VAFTDTERLIGDAAKNQVALNPQN 65 | 112 KAFYPEEIVTNAVITVPA 119 139 148

HSP60 14 VSRVLAPHLTRA 25 | 66 QSWGSPKVTKDGV 78 | 129 EKISKGANPVEIRRGVMLAVD 149 | 222 GYISPYFINTS 232

SPEK **KEQFNYSD** GGIIKTSDVKPEEKAIYINIFGEKELRTLTAKDKITFKNNIVTL **QEIDVRLRKSLMGDSKIKLYEYDSLYKKGFWDIH** YKDGGIRHTNLFTYPDYTDNETIDMSKVSHFDVHLNEDFSKD
(position marks: 140, 150, 160, 170, 180, 190, 200, 210, 220, 230, 240, 250)

Grp94 354 EYKAFYKSFSKESD 367 | 375 FTAEGEVTFKS 385 | 556 ERLLKKGYEVIY 567 | 707 DKTVLDLAVV 716

HSP90 PEYLNF 384 | 656 DKSVKDLVILLYETALLSSGFSLE 679

Grp78 345 VLEDSDLK 352 | 407 GDQDTGDLVLLDVCPLTLGIETV 429 | 550 EEDKKLKERI 559 | 563 NELESYAYS 571 | 573 KNQIGDKEKLGGKL 586 | 590 DKETMEKA 597

HSP70 507 KGRLSKEEI 515

HSP60 323 AVFGEEGL 330 | 349 IVTKDDAMLLKQIEKRIQEII 359 366 375 | 396 KLSDGVAVLKVG 407 | 411 DVEVNEKKDRVTDALNFVNMVEKGIIDPTKV 426 510 524
