# Supplementary material for: Two homologous sequences of Grp78 and HSP70 represent tumor antigens shared with streptococcal superantigens in eliciting an antitumor immune response: an immunoinformatic investigation
Source: Front Immunol. 2025 Sep 11;16:1644687. doi: 10.3389/fimmu.2025.1644687 (PMC12460249; doi:10.3389/fimmu.2025.1644687)
Supplement: Supplementary Figure 3 — Predicted MHC-I binding peptides of HSPs matching exotoxin epitopes. MHC-I epitopes of HSPs (supplemental Table 2) overlapping exotoxin epitopes (supplemental Table 1) are shown based on alignments (supplemental Figure 2). Bold amino acids represent MHC-I peptides; non-bold are adjacent residues. Epitope numbers refer to alignment positions; bold residues adjacent to numbered epitopes refer to epitope residues that may or may not match known exotoxin or HSP epitopes. Multiple epitopes often exist within a single MHC-I peptide. [file DataSheet3.pdf]

Supplemental Figure 3

Protein	Start	Sequence	End	allele	IC50
SPEA	6	KVLKKMVFF	14	B*57:01	321.77
HSP70	60	ALNPQNTVF	68	B*15:01	14.86
SPEA	44	LVKNLQNIYFLY	52	B*15:01	195.71
HSP70	126	VLTKMKEIAEAY	134	B*15:01	79.40
SPEA	66	KSVDQLLSHDLIY	78	A*01:01	293.81
Grp94	322	KTKKVEKTVW	331	B*57:01	8.66
*	322	KTKKVEKTVWDW	333	B*57:01	17.44
*	325	KVEKTVWDW	333	B*57:01	125.40
SPEA	173	QELDYKVRKYLTDNK	182	B*44:02	100.20
HSP90	454	QNRKKLSELL	463	B*08:01	160.24
SPEA	187	KQLYTNGPSKYET	196	A*03:01	8.75
	188	QLYTNGPSKYET	196	A*03:01	16.62
	188	QLYTNGPSKYET	197	B*15:01	38.33
	196	KYETGYIKF	204	A*24:02	58.72
Grp94	537	KIYFMAGSSRKEA	547	A*03:01	17.64
Grp78	556	KKLKERIDTRNEL	565	B*40:01	128.25
* HSP70	533	RERVSAKNAL	542	B*40:01	58.29
	537	SAKNALESY	545	B*15:01	106.78
SPEC	80	SSEMSYEASQKFKR	90	B*44:02	10.37
	80	MSYEASQKF	90	B*15:01	63.50
				B*57:01	39.45
HSP60	275	AEDVDGEALSTL	283	B*40:01	4.25
	280	GEALSTLVL	288	B*40:01	5.17
SPEC	95	HVDVFGLFY	103	A*01:01	3.61
	104	ILNSHTGEY	112	B*15:01	47.30
Grp94	356	EEDEYKAFYKSFSKESD	364	A*03:01	18.36
SPEC	157	FQEIDFKIRKYLMD	166	B*44:02	40.87
HSP70	474	IEVTFDIDANGIL	486	B*40:01	190.68
* Grp78	497	IEVTFEIDVNGIL	509	B*40:01	107.78
	500	TFEIDVNGIL	509	B*40:01	11.57
	501	FEIDVNGIL	509	B*40:01	2.83
SPEC	165	KYLMDNYKI	173	A*24:02	87.81
	166	YLMDNYKIYDA	174	B*15:01	65.71
HSP70	484	GILNVTATDK	493	A*03:01	89.33

Protein	Start	Sequence	End	allele	IC50
SPEM	75	YALIKSYSVI	83	B*08:01	9.87
Grp94	356	KAFYKSFSK	364	A*03:01	18.36
HSP90	288	EELNKTKPIW	297	B*44:02	79.33
SPEM	79	KSYSVICKEQYNY	89	B*57:01	477.56
Grp94	364	KESDDPMAY	372	B*44:02	68.91
	363	SKESDDPMAY	372	B*44:02	272.78
SPEM	99	TSDREKLDSTIY	110	A*01:01	228.38
HSP90	454	SQNRKKLSELL	463	B*08:01	160.24
SPEM	125	KYKITFQNRF	134	A*24:02	31.59
Grp94	479	KYNDTFWKEF	488	A*24:02	39.50
SPEM	128	KITFQNRFVTFQEI	137	B*57:01	97.69
	129	TFQNRFVTFQEI	137	A*24:02	29.26
	130	FQNRFVTF	137	B*15:01	119.50
				B*08:01	417.76
Grp94	486	DTFWKEFGTNIKLGV	494	B*40:01	5.17
* HSP90	438	YEQFSKNIKLGI	447	B*40:01	49.97
HSP60	236	KCEFQDAYVLLSE	245	B*40:01	75.55
	237	CEFQDAYVLLSE	245	B*40:01	15.23
SPEM	148	RKSLMSDNRIKLYEH	157	A*01:01	164.05
	149	MSDNRIKLY	157	A*01:01	14.11
Grp78	556	KERIDTRNEL	565	B*40:01	128.25
* HSP70	533	RERVSAKNAL	542	B*40:01	58.29
	537	SAKNALESY	545	B*15:01	106.78
SPEM	155	KLYEHNSICK	164	A*03:01	10.14
	155	KLYEHNSICKK	165	A*03:01	39.28
HSP60	396	KLSDGVAVLKV	404	A*02:01	14.87
	396	KLSDGVAVLKV	405	A*03:01	19.77
SPEM	165	ICKKGYWGIHYK	173	A*03:01	14.06
HSP70	569	KVLDKCQEVISW	580	B*57:01	495.92
SPEK	42	APRYDKDEI	50	B*07:02	41.47
HSP90	189	HLKEDQTEY	197	B*15:01	263.24
SPEK	125	KSYHVVCKEQF	135	B*57:01	320.30
HSP90	374	CEELIPEYL	382	B*40:01	56.95
SPEK	201	KLYEYDSLYK	210	A*03:01	5.82
	201	KLYEYDSLYKK	211	A*03:01	35.58
HSP60	396	KLSDGVAVLKV	404	A*02:01	14.87
	396	KLSDGVAVLKV	405	A*03:01	19.77
