# Supplementary material for: Two homologous sequences of Grp78 and HSP70 represent tumor antigens shared with streptococcal superantigens in eliciting an antitumor immune response: an immunoinformatic investigation
Source: Front Immunol. 2025 Sep 11;16:1644687. doi: 10.3389/fimmu.2025.1644687 (PMC12460249; doi:10.3389/fimmu.2025.1644687)
Supplement: Supplementary Figure 4 — Alignment of Grp78, HSP70, and 5T4. T-Coffee alignment highlights the regions with higher sequence consensus, excluding the first 73 and last 4 amino acids of 5T4 due to low similarity. An overall similarity score of 945 was found among the three proteins, with HSP70 showing the highest sequence consensus (960). Symbols indicate identical (*, red), conserved (:), semi-conserved (.) residues. Bold indicates shared MHC-I epitopes in Grp78 and HSP70 ( Table 1 ). [file DataSheet4.pdf]

# Supplementary Figure S4

```
        157          170                 190               208  222                 243  341               360                 380
Grp78  --AEAYLGKKVTHAVVTVPAYFNDAQRQATKDAGTIAGLNVMRIINEPTAAAIA---VFDLGGGTFDVSLLTIDNGVFE---PVQKVLEDSDLKKSDIDEIVLVGGSTRIPKIQQLVKEFFNGKEPSRGINPD
        131                150                 170         182  197                 218  316                   340                  360
HSP70  --AEAYLGYPVTNAVITVPAYFNDSQRQATKDAGVIAGLNVLRIINEPTAAAIA---IFDLGGGTFDVSILTIDDGIFE---PVEKALRDAKLDKAQIHDLVLVGGSTRIPKVQKLLQDFFNGRDLNKSINPD
        74              90                  110            121  122     131  132    139  142              160                 180    187 188
5T4    --TVKCVNRNLTEVPTDLPAYVRNLFLTGNQLAVLPAGAFARR----PPLAELA---ALNLSGSRLD----EVRAGAFE---PSLRQLDLSHNPLADLSPFAFSGSNASVSAPSPLVELILNHIVPPE--DER
         :   :.  :*..   :***..:    ..: *   **  . *    *. * :*    ::*.*. :*     :  * **   *  : *  :.   :::  :.: *..: :.  . *:: ::*     .  :

              400                  420                  440                 460                 480                 500                 520
Grp78  EAVAYGAAVQAGVLSGDQDT--GDLVLLDVCPLTLGIETVGGVMTKLIPRNTVVPTKKSQIFSTASDNQPTVTIKVYEGERPLTKDNHLLGTFDLTGIPPAPRGVPQIEVTFEIDVNGILRVTAEDKGTGNKN
                   380                 400                 420                 440                 460                 480
HSP70  EAVAYGAAVQAAILMGDKSENVQDLLLLDVAPLSLGLETAGGVMTALIKRNSTIPTKQTQIFTTYSDNQPGVLIQVYEGERAMTKDNNLLGRFELSGIPPAPRGVPQIEVTFDIDANGILNVTATDKSTGKAN
                         210               228  229                   251    252                   274 275             293 294           310
5T4    QNRSFEGMVVAALLAGRALQGLRRLELASNHFLYLPRD----VLAQLPSLRHLDLSNNSLVSLTY------VSFRNLTHLESLHLEDNALKVLH-NGTLAELQGLPHIRVFLDN-NPWVCDCHMADMVTWLK-
       : ::  . * *.:* *        * * .   * *  :    *:: *   .    :::: :  *       * ::     ..:  ::: *  :. .*  .  :*:*:*.* ::     :      *  *

                       540                 560                 580                 600                 620             636
Grp78  KITITNDQNRLTPEEIERMVNDAEKFAEEDKKLKERIDTRNELESYAYSLKNQIGDKEKLGGKLSSEDKETMEKAVEEKIEWLESHQDADIEDFKAKKKELEEIVQPIISKLYG--
       500                520                 540                  560                 580                 600         612
HSP70  KITITNDKGRLSKEEIERMVQEAEKYKAEDEVQRERVSAKNALESYAFNMKSAVED-EGLKGKISEADKKKVLDKCQEVISWLDANTLAEKDEFEHKRKELEQVCNPIISGLYQ--
       311               330                 350      359   360         372 373              390                 410   416
5T4    ETEVVQGKDRLTCAYPEKMRNRVLLELNSADLDCDPILPPSLQTSYVFL-----GIVLALIGAIFLL---VLYLNRKGIKKWMHNIRDACRDHMEGYHYRYEINADPRLTNLSS--
       :  :.:.:.**:    *:* : .     . .   : : . .   **.:           * * :       :    :   .*:.    *  :.::  : . *   :* :: *
```
