# Supplementary material for: Two homologous sequences of Grp78 and HSP70 represent tumor antigens shared with streptococcal superantigens in eliciting an antitumor immune response: an immunoinformatic investigation
Source: Front Immunol. 2025 Sep 11;16:1644687. doi: 10.3389/fimmu.2025.1644687 (PMC12460249; doi:10.3389/fimmu.2025.1644687)
Supplement: Supplementary Table 1 — Predicted best MHC-I peptides in exotoxins. Exotoxins were scanned for MHC-I peptides using IEDB (see Methods) with a set of common HLA–A, HLA–B alleles. IC50 values <500 nM (in parentheses) was considered for good binding. [file DataSheet5.pdf]

Supplemental Table 1

exotoxin	A*01:01	A*02:01	A*03:01	A*24:02	B*07:02	B*08:01	B*15:01	B*40:01	B*44:02	B*57:01
SPEA	66-78 (293.81)<br>105-113 (24.97)<br>105-114 (24.42)<br>183-190 (18.05)<br>236-247 (104.91)		187-196 (8.75)<br>188-196 (16.62)	108-116 (43.77)<br>196-204 (58.72)			44-52 (195.71)<br>188-197 (38.33)<br>223-231 (41.97)		173-182 (100.20)	6-14 (321.77)
SPEC	95-103 (3.61)	112-120 (6.46)	172-180 (31.80)	165-173 (87.81)	202-212 (56.98)<br>178-187 (33.11)		82-90 (63.50)<br>104-112 (47.30)<br>166-174 (65.71)<br>172-180 (17.01)		80-90 (10.37)<br>157-166 (40.87)	82-90 (39.45)
SPEM	99-110 (228.38)<br>148-157 (164.05)<br>149-157 (14.11)<br>179-188 (4.76)		22-30 (42.04)<br>155-164 (10.14)<br>155-165 (39.28)<br>165-173 (14.06)	129-137 (29.26)<br>125-134 (31.59)<br>170-179 (310.88)		75-83 (9.87)<br>130-137 (417.76)<br>141-148 (74.98)	130-137 (119.50)	68-77 (28.42)		36-46 (497.73)<br>38-46 (453.56)<br>79-89 (477.56)<br>128-137 (97.69)
SPEK	19-29 (38.74)<br>20-29 (17.76)	182-190 (132.40)	201-210 (5.82)<br>201-211 (35.58)		42-50 (41.47)	187-194 (74.98)	17-25 (12.64)<br>201-209 (91.57)			125-135 (320.30)
