# Supplementary material for: Two homologous sequences of Grp78 and HSP70 represent tumor antigens shared with streptococcal superantigens in eliciting an antitumor immune response: an immunoinformatic investigation
Source: Front Immunol. 2025 Sep 11;16:1644687. doi: 10.3389/fimmu.2025.1644687 (PMC12460249; doi:10.3389/fimmu.2025.1644687)
Supplement: Supplementary Table 2 — Predicted best MHC-I peptides in HSPs. HSPs were scanned for MHC-I peptides using IEDB (see Methods) with a set of common HLA–A, HLA–B alleles. IC50 <500 nM (in parentheses) was considered for good binding. [file DataSheet6.pdf]

Supplemental Table 2

HSPs	A*01:01	A*02:01	A*03:01	A*24:02	B*07:02	B*08:01	B*15:01	B*40:01	B*44:02	B*57:01
Grp94	518-527 (13.65)<br>668-678 (18.50)		87-95 (15.62)<br>356-364 (18.36)<br>455-467 (15.35)<br>537-547 (17.64)	479-488 (39.50)<br>677-685 (28.18)		85-93 (9.02)<br>424-432 (197.60)	250-258 (495.05)<br>272-280 (38.87)<br>667-677 (497.29)<br>727-735 (92.80)	253-261 (47.88)<br>379-387 (5.21)<br>448-456 (95.08)<br>486-494 (5.17)<br>685-693 (4.68)	364-372 (68.91)<br>612-621 (36.17)<br>613-621 (16.44)	322-331 (8.66)<br>322-333 (17.44)<br>325-333 (125.40)
HSP90	52-61 (54.69)<br>304-313 (48.15)<br>618-627 (64.75)		410-418 (59.26)<br>573-581 (36.97)<br>641-649 (470.14)	433-441 (20.50)	81-89 (47.79)<br>343-351 (14.66)	454-463 (160.24)	132-142 (141.74)<br>189-197 (263.24)<br>483-492 (56.55)<br>520-528 (26.91)<br>618-627 (53.88)	24-32 (7.22)<br>374-382 (56.95)<br>438-447 (49.97)<br>534-542 (97.59)	24-32 (66.08)<br>288-297 (79.33)<br>428-437 (133.56)<br>485-493 (130.00)	
Grp78		236-244 (14.77)<br>261-269 (39.00)<br>415-424 (28.25)	261-271 (35.19)<br>268-276 (6.54)<br>464-474 (79.36)		470-478 (150.60)<br>490-499 (37.38)<br>490-501 (278.80)		30-39 (40.22)<br>151-160 (44.78)<br>152-160 (43.38)	255-263 (292.01)<br>290-299 (111.74)<br>313-322 (16.19)<br>500-509 (11.57)<br>501-509 (2.83)<br>556-565 (128.25)	319-327 (57.36)<br>594-604 (269.64)	594-604 (291.86)<br>596-604 (28.88)

HSPs	A*01:01	A*02:01	A*03:01	A*24:02	B*07:02	B*08:01	B*15:01	B*40:01	B*44:02	B*57:01
HSP70	431-443<br>(15.61)	209-219<br>(22.31)<br>211-219<br>(7.33)	484-493<br>(89.33)		467-476<br>(37.80)<br>467-478<br>(278.80)		6-15<br>(20.21)<br>60-68<br>(14.86)<br>126-134<br>(79.40)<br>423-431<br>(48.18)<br>517-525<br>(210.59)<br>537-545<br>(106.78)	474-486<br>(14.79)<br>533-542<br>(58.29)		82-90<br>(103.53)<br>569-580<br>(495.92)
HSP60	377-385<br>(15.84)	17-25<br>(78.79)<br>389-397<br>(37.47)<br>396-404<br>(14.87)<br>528-538<br>(251.86)	16-24<br>(71.81)<br>344-352<br>(141.79)<br>396-405<br>(19.77)		12-22<br>(90.01)	265-273<br>(89.56)	17-26<br>(33.24)<br>234-243<br>(11.88)<br>495-503<br>(42.94)<br>529-538<br>(43.98)	236-245<br>(75.55)<br>237-245<br>(15.23)<br>275-283<br>(4.25)<br>280-288<br>(5.17)<br>326-334<br>(7.86)		24-32<br>(82.90)<br>58-68<br>(21.86)<br>59-68<br>(19.06)<br>60-68<br>(5.23)
