# Supplementary material for: Two homologous sequences of Grp78 and HSP70 represent tumor antigens shared with streptococcal superantigens in eliciting an antitumor immune response: an immunoinformatic investigation
Source: Front Immunol. 2025 Sep 11;16:1644687. doi: 10.3389/fimmu.2025.1644687 (PMC12460249; doi:10.3389/fimmu.2025.1644687)
Supplement: Supplementary Table 3 — Predicted best MHC-II peptides in exotoxins and HSPs. MHC-II peptides in exotoxins and Grp94, Grp78, HSP70 were predicted using IEDB (2023.05 method) with seven prevalent HLA alleles. Only top binders (percentile rank ≤0.5) were included. [file DataSheet7.pdf]

Supplemental Table 3

	HLA-DRB1*03:01	HLA-DRB1*07:01	HLA-DRB1*15:01	HLA-DRB3*01:01	HLA-DRB3*02:02	HLA-DRB4*01:01	HLA-DRB5*01:01
**SPEA**	179 VRKYLTDNKQLYTNG 193<br>178 KVRKYLTDNKQLYTN 192		225 SKYLMIYKDNETLDS 239<br>224 QSKYLMIYKDNETLD 238<br>226 KYLMIYKDNETLDSN 240	179 VRKYLTDNKQLYTNG 193	73 SHDLIYNNSGPNYDK 87<br>72 LSHDLIYNNSGPNYD 86<br>74 HDLIYNNSGPNYDKL 88<br>71 LLSHDLIYNNSGPNY 85	167 KKMVTAQELDYKVRK 181<br>166 NKKMVTAQELDYKVR 180<br>26 QEVFAQQDPDPSQLH 40<br>25 SQEVFAQQDPDPSQL 39	82 GPNYDKLKTELKNQE 96<br>81 GPNYDKLKTELKNSQ 95<br>197 YETGYIKFIPKNKES 211<br>198 ETGYIKFIPKNKESF 212<br>196 KYETGYIKFIPKNKE 210<br>80 VSGPNYDKLKTELKN 94<br>83 PNYDKLKTELKNQEM 97
**SPEC**	61 THTLNIDTQKYRGKD 75<br>60 TTHTLNIDTQKYRGK 74<br>22 SPIIKSDSKKDISNV 36<br>21 ISPIIKSDSKKDISN 35<br>62 HTLNIDTQKYRGKDY 76<br>59 STTHTLNIDTQKYRG 73<br>20 TISPIIKSDSKKDIS 34<br>23 PIIKSDSKKDISNVK 37	55 RVNFSTTHTLNIDTQ 69<br>54 CRVNFSTTHTLNIDT 68<br>53 DCRVNFSTTHTLNID 67<br>56 VNFSTTHTLNIDTQK 70<br>114 YGGITPAQNNKVNHK 128	114 YGGITPAQNNKVNHK 128	86 ASQKFKRDDHVDVFG 100<br>87 SQKFKRDDHVDVFGL 101<br>85 EASQKFKRDDHVDVF 99		151 KDIVTFQEIDFKIRK 165<br>150 EKDIVTFQEIDFKIR 164	
**SPEM**	145 RKSLMSDNRIKLYEH 159<br>144 LRKSLMSDNRIKLYE 158<br>143 RLRKSLMSDNRIKLY 157<br>146 KSLMSDNRIKLYEHN 160	47 DDDNYNKVISSNVSP 61<br>48 DDNYNKVISSNVSPA 62<br>86 KEQYNYVDGGLIRTS 100<br>85 CKEQYNYVDGGLIRT 99<br>46 WDDDNYNKVISSNVS 60<br>84 LCKEQYNYVDGGLIR 98<br>49 DNYNKVISSNVSPAQ 63	151 DNRIKLYEHNSICKK 165		52 NKVISSNVSPAQERR 66<br>31 IGTQLIFNTNEKTRV 45<br>32 GTQLIFNTNEKTRVW 46<br>51 YNKVISSNVSPAQER 65<br>30 KIGTQLIFNTNEKTR 44<br>53 KVISSNVSPAQERRF 67	132 NRFVTFQEIDVRLRK 146	
**SPEK**	190 LRKSLMGDSKIKLYE 204<br>191 RKSLMGDSKIKLYEY 205<br>189 RLRKSLMGDSKIKLY 203	92 NDNTFKKVISSNLSP 106<br>93 DNTFKKVISSNLSPS 107<br>132 KEQFNYSDGGIIKTS 146<br>91 WNDNTFKKVISSNLS 105<br>131 CKEQFNYSDGGIIKT 145<br>116 DHVNIFAIVKSYHVV 130<br>130 VCKEQFNYSDGGIIK 144<br>118 VNIFAIVKSYHVVCK 132<br>94 NTFKKVISSNLSPSQ 108<br>117 HVNIFAIVKSYHVVC 131		79 QLIFNTDENTTVWND 93<br>78 TQLIFNTDENTTVWN 92<br>77 GTQLIFNTDENTTVW 91	173 KITFKNNIVTLQEID 187<br>96 FKKVISSNLSPSQER 110<br>97 KKVISSNLSPSQERN 111<br>62 KEIIEKNNISINAKQ 76<br>172 DKITFKNNIVTLQEI 186	178 NNIVTLQEIDVRLRK 192<br>177 KNNIVTLQEIDVRLR 191	161 GEKELRTLTAKDKIT 175<br>162 EKELRTLTAKDKITF 176

	HLA-DRB1*03:01	HLA-DRB1*07:01	HLA-DRB1*15:01	HLA-DRB3*01:01	HLA-DRB3*02:02	HLA-DRB4*01:01	HLA-DRB5*01:01
**Grp94**	746 SLNIDPDAKVEEEPE 760<br>724 RSGYLLPDTKAYGDR 738<br>725 SGYLLPDTKAYGDRI 739<br>744 RLSLNIDPDAKVEEE 758<br>745 LSLNIDPDAKVEEEP 759<br>723 LRSGYLLPDTKAYGD 737<br>747 LSLNIDPDAKVEEEP 761<br>726 GYLLPDTKAYGDRIE 740<br>229 EFSVIADPRGNTLGR 243<br>743 LRLSLNIDPDAKVEE 757<br>228 NEFSVIADPRGNTLG 242<br>593 KEGVKFDESEKTKES 607		194 QFGVGFYSAFLVADK 208	744 RLSLNIDPDAKVEEE 758<br>743 LRLSLNIDPDAKVEE 757<br>593 KEGVKFDESEKTKES 607<br>592 AKEGVKFDESEKTKE 606<br>742 MLRLSLNIDPDAKVE 756<br>745 LSLNIDPDAKVEEEP 759	156 REELVKNLGTIAKSG 170<br>155 TREELVKNLGTIAKS 169<br>154 MTREELVKNLGTIAK 168	630 KIEKAVVSQRLTESP 644<br>632 EKAVVSQRLTESPCA 646<br>631 IEKAVVSQRLTESPC 645	535 QDKIYFMAGSSRKEA 549<br>536 DKIYFMAGSSRKEAE 550<br>534 KQDKIYFMAGSSRKE 548<br>612 EKEFEPLLNWMKDKA 626<br>611 VEKEFEPLLNWMKDK 625<br>537 KIYFMAGSSRKEAES 551<br>73 SEKFAFQAEVNRMMK 87
**Grp94**	385 SRGINPDEAVAYGAA 399<br>275 GKDVRKDNRAVQKLR 289<br>273 KTGKDVRKDNRAVQK 287<br>274 TGKDVRKDNRAVQKL 288<br>384 PSRGINPDEAVAYGA 398<br>386 RGINPDEAVAYGAAV 400<br>383 EPSRGINPDEAVAYG 397<br>276 KDVRKDNRAVQKLRR 290<br>49 RVEIIANDQGNRITP 63<br>50 VEIIANDQGNRITPS 64<br>469 ERPLTKDNHLLGTFD 483<br>468 GERPLTKDNHLLGTF 482		460 TVTIKVYEGERPLTK 474<br>459 PTVTIKVYEGERPLT 473	384 PSRGINPDEAVAYGA 398<br>385 SRGINPDEAVAYGAA 399<br>50 VEIIANDQGNRITPS 64<br>383 EPSRGINPDEAVAYG 397<br>49 RVEIIANDQGNRITP 63<br>386 RGINPDEAVAYGAAV 400<br>51 EIIANDQGNRITPSY 65<br>496 QIEVTFEIDVNGILR 510<br>48 GRVEIIANDQGNRIT 62<br>497 IEVTFEIDVNGILRV 511<br>498 EVTFEIDVNGILRVT 512<br>382 KEPSRGINPDEAVAY 396<br>172 VPAYFNDAQRQATKD 186<br>499 VTFEIDVNGILRVTA 513		47 NGRVEIIANDQGNRI 61<br>46 KNGRVEIIANDQGNR 60<br>45 FKNGRVEIIANDQGN 59<br>48 GRVEIIANDQGNRIT 62<br>535 PEEIERMVNDAEKF 549<br>506 NGILRVTAEDKGTGN 520<br>534 TPEEIERMVNDAEKF 548<br>96 KRLIGRTWNDPSVQQ 110<br>447 KSQIFSTASDNQPTV 461<br>505 VNGILRVTAEDKGTG 519<br>229 TFDVSLLTIDNGVFE 243<br>95 AKRLIGRTWNDPSVQ 109<br>446 KKSQIFSTASDNQPT 460<br>533 LTPEEIERMVNDAEK 547<br>569 AYSLKNQIGDKEKLG 583	463 IKVYEGERPLTKDNH 477<br>111 DIKFLPFKVVEKKTK 125<br>462 TIKVYEGERPLTKDN 476<br>110 QDIKFLPFKVVEKKT 124<br>464 KVYEGERPLTKDNHL 478<br>505 VNGILRVTAEDKGTG 519<br>356 IDEIVLVGGSTRIPK 370

	HLA-DRB1*03:01	HLA-DRB1*07:01	HLA-DRB1*15:01	HLA-DRB3*01:01	HLA-DRB3*02:02	HLA-DRB4*01:01	HLA-DRB5*01:01
**HSP70**	360 NKSINPDEAVAYGAA 374<br>359 LNKSINPDEAVAYGA 373<br>358 DLNKSINPDEAVAYG 372<br>361 KSINPDEAVAYGAAV 375<br>25 KVEIIANDQGNRTTP 39	39 PSYVAFTDTERLIGD 53<br>38 TPSYVAFTDTERLIG 52	437 GVLIQVYEGERAMTK 451<br>436 PGVLIQVYEGERAMT 450<br>438 VLIQVYEGERAMTKD 452<br>435 QPGVLIQVYEGERAM 449	359 LNKSINPDEAVAYGA 373<br>360 NKSINPDEAVAYGAA 374<br>358 DLNKSINPDEAVAYG 372<br>361 KSINPDEAVAYGAAV 375<br>475 EVTFDIDANGILNVT 489<br>474 IEVTFDIDANGILNV 488<br>473 QIEVTFDIDANGILN 487<br>357 RDLNKSINPDEAVAY 371<br>146 VPAYFNDSQRQATKD 160	411 TALIKRNSTIPTKQT 425<br>410 MTALIKRNSTIPTKQ 424<br>56 KNQVALNPQNTVFDA 70	318 EKALRDAKLDKAQIH 332<br>317 VEKALRDAKLDKAQI 331<br>23 HGKVEIIANDQGNRT 37<br>316 PVEKALRDAKLDKAQ 330<br>483 NGILNVTATDKSTGK 497<br>22 QHGKVEIIANDQGNR 36<br>24 GKVEIIANDQGNRTT 38<br>319 KALRDAKLDKAQIHD 333<br>482 ANGILNVTATDKSTG 496<br>21 FQHGKVEIIANDQGN 35<br>484 GILNVTATDKSTGKA 498<br>481 DANGILNVTATDKST 495<br>204 TFDVSILTIDDGIFE 218<br>315 EPVEKALRDAKLDKA 329	440 IQVYEGERAMTKDNN 454<br>522 AEKYKAEDEVQRERV 536<br>521 EAEKYKAEDEVQRER 535<br>439 LIQVYEGERAMTKDN 453<br>290 GIDFYTSITRARFEE 304<br>38 TPSYVAFTDTERLIG 52<br>441 QVYEGERAMTKDNNL 455<br>523 EKYKAEDEVQRERVS 537<br>520 QEAEKYKAEDEVQRE 534<br>438 VLIQVYEGERAMTKD 452<br>289 EGIDFYTSITRARFE 303<br>37 TTPSYVAFTDTERLI 51
